# Supplementary material for: Cortical State Fluctuations during Sensory Decision Making
Source: Curr Biol. 2020 Dec 21;30(24):4944–4955.e7. doi: 10.1016/j.cub.2020.09.067 (PMC7758730; doi:10.1016/j.cub.2020.09.067)
Supplement: Document S1. Figures S1–S7 [file mmc1.pdf]

Current Biology, Volume *30*

Supplemental Information

Cortical State Fluctuations during Sensory Decision Making

Elina A.K. Jacobs, Nicholas A. Steinmetz, Andrew J. Peters, Matteo Carandini, and Kenneth D. Harris

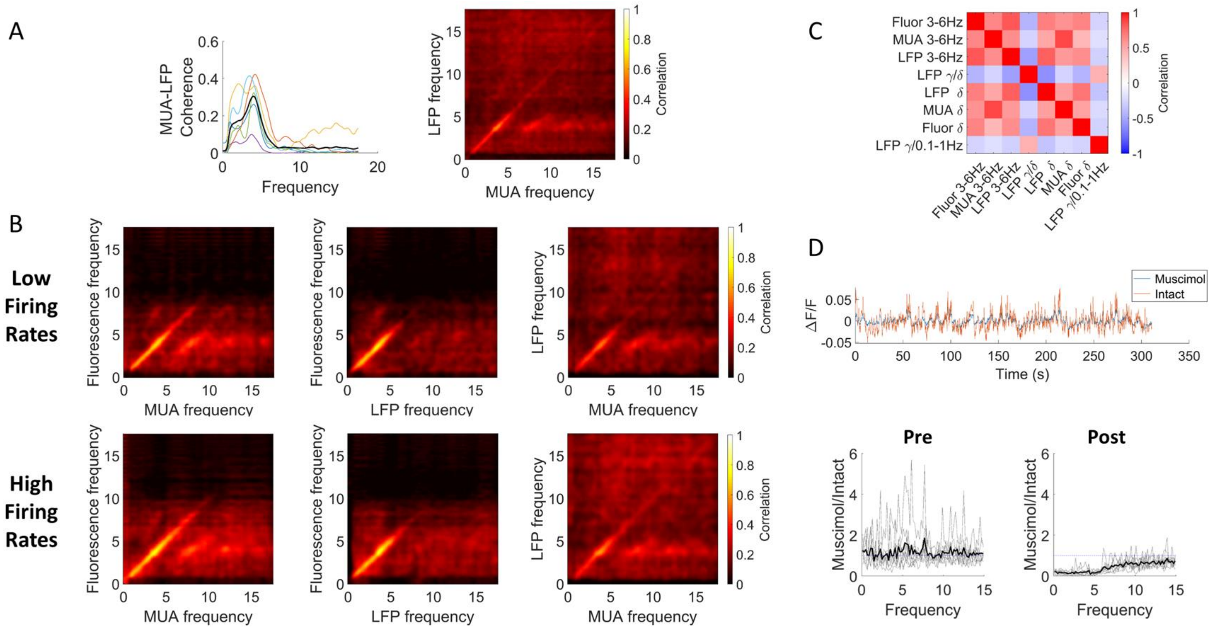

Figure S1. Low-frequency widefield power correlations with electrophysiological measures of power. Related to Figure 2. A. Left: coherence correlation between MUA and LFP. Black trace = average, colored traces = individual experiments (n = 6 experiments from 6 animals). Coherence of LFP with MUA is smaller than coherence of widefield fluorescence with MUA (peak of 0.31 vs. 0.56; $p<0.05$, t-test; cf. Figure 2C), likely reflecting non-local contributions to the LFP signal. Right: cross-frequency power correlation between MUA and LFP. **B.** Power measured by widefield imaging is strongly correlated with power measured by electrophysiology, and does not reflect changes in firing rate. We divided each dataset into low and high firing rate periods (epochs of 2 s or longer consistently above or below the median MUA firing rate). The correlations between widefield and MUA power, widefield and LFP power, and MUA and LFP power all remain comparable when restricted to periods of low (top row) and high (bottom row) firing rates. **C.** Correlations between widefield fluorescence, MUA, and LFP signals across multiple frequency bands, together with LFP power ratios (gamma to delta; gamma to ultraslow). Each square represents the average correlation across six experiments. Note the positive correlation of power in all low-frequency bands and modalities, and its inverse correlation to the LFP γ/δ and γ/infraslow ratios (sometimes used as measure of desynchronization). δ = 1-4Hz, γ = 20-80Hz. **D.** Local application of muscimol ablates low frequency power in the widefield signal. Top: Example widefield trace of cortical activity with and without muscimol application. Blue trace indicates the signal from the muscimol-treated cortical region, brown trace indicates the signal from the corresponding contralateral untreated cortical region. Bottom: ratio of muscimol-treated to control (intact) power spectra. Left: Before muscimol application, the ratio hovers around 1, indicating that the power spectra from the two cortical regions are comparable across frequencies. Right: After muscimol treatment, the ratio drops to close to zero at frequencies below ~8Hz, indicating a large drop in widefield power after local cortical activity has been inhibited by muscimol, indicating that the majority of the widefield signal reflects local spiking activity.

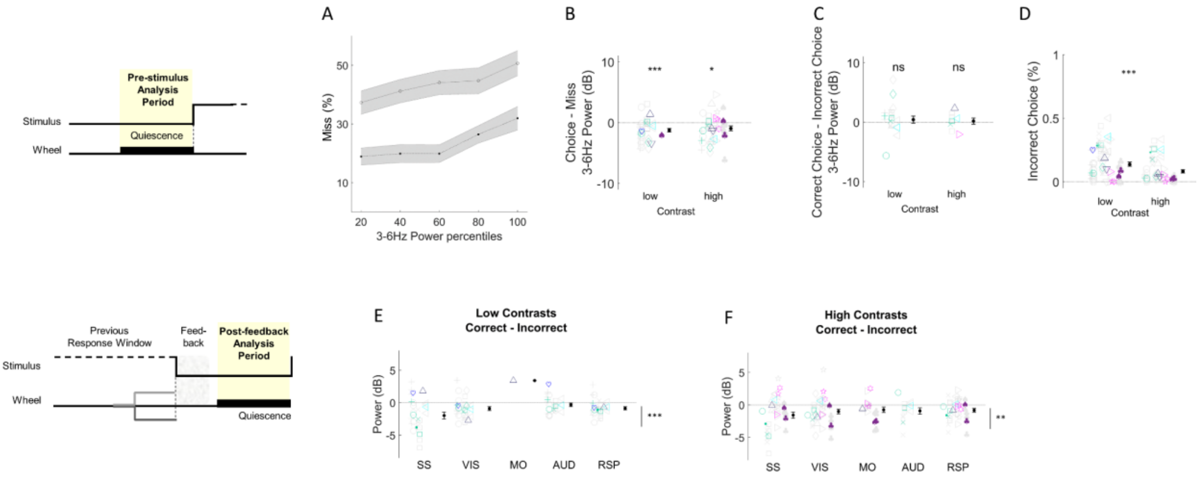

Figure S2. Power differences between trial types do not depend on contrast. Related to Figures 2 and 6. **A-D:** Behavior and power differences per contrast condition. **A.** Average percent Miss trials in different 3-6Hz power percentiles for high (filled circles) and low (open circles) contrasts. Lower contrasts are generally more likely to be 'Missed', but high contrasts also get Missed, and increasing 3-6Hz power equally increases the Miss probability for both low and high contrasts ($p<0.001$ for both high and low contrasts, generalized linear mixed-effects model). **B**. Summary of 3-6Hz power difference in visual cortex between choice and Miss trials for low and high contrasts. Choice trials are significantly more desynchronized for both contrast conditions (condition main effect $p<0.001$; contrast main effect $p>0.05$, nested mixed effects ANOVA). **C**. Same as B but comparing Correct and Incorrect Choice trials (condition main effect $p>0.05$; contrast main effect $p>0.05$, nested mixed effects ANOVA). **D**. Summary of percent Incorrect Choice for low and high contrasts across experiments. Animals are significantly more likely to provide an Incorrect Choice for low contrasts ($p<0.001$, Wilcoxon signed rank test). High contrasts consist of trials including and above 50% contrast, low contrast trials consist of trials below 50% contrast. Contrast comparison trials were included in the high contrast trials as excluding them did not affect the results (data not shown). **E-F:** The effect of reward does not depend on contrast. **E.** Summary difference in 3-6Hz power after Correct and Incorrect low contrast trials. **F.** As E but for high contrast trials. After both low and high contrasts, Correct i.e. rewarded trials show prolonged desynchronization lasting into the quiescent period of the following trial. There was no effect of contrast on the power difference between rewarded (Correct) and non-rewarded (Incorrect) trials ($p<0.05$, nested mixed ANOVA where session was nested into mouse, session was set a random effect, and the main effects and interactions of session, ROI, response (Correct or Incorrect) and subject were included in the model). ***, $p < 0.001$; ns, $p \geq 0.05$

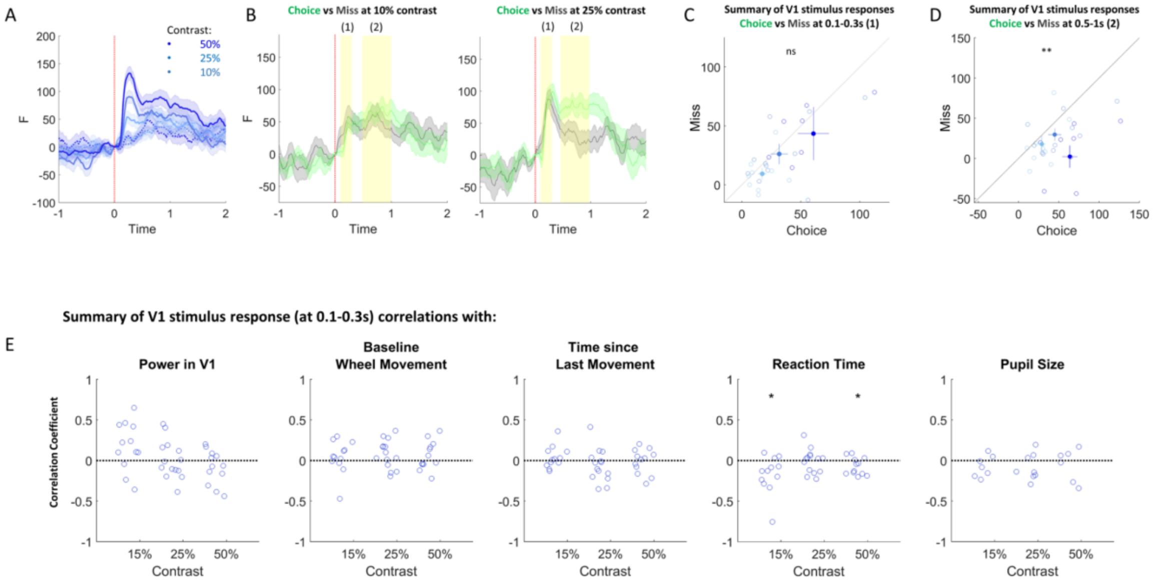

Figure S3. V1 stimulus responses measured by widefield imaging. Related to Figure 2. A. Time course of V1 responses to visual stimuli of different contrasts from an example mouse. **B**. Example time courses for 10% and 25% contrast, split by behavioral condition (Choice and Miss). **C**. Population summary of average amplitudes of V1 responses at 0.1 to 0.3 seconds (highlighted in B as (1)) post stimulus onset. The amplitude was computed as the average fluorescence signal obtained from the time period in (1). Open circles indicate individual datasets, filled circles indicate average per contrast. Colors are the same as indicated in the legend in A. There was no significant difference between Choice and Miss responses ($p>0.05$, 2-way ANOVA). **D**. Same as C but for a later timepoint (0.5 to 1 seconds post stimulus onset) that overlaps with reaction time onset. **E**. Summary of correlations between V1 amplitudes at the first timepoint (1) and relevant physiological and behavioral factors. Shaded areas in A and B indicated standard error of the mean (SEM); bars in C and D indicate SEM. n = 14 experiments from 4 mice. Only datasets in which no movement was detected during the first analysis window were included, and only contrast conditions with at least 5 trials per behavioral condition were included for each dataset. *, $p < 0.05$; **, $p<0.01$; ns, $p>0.05$

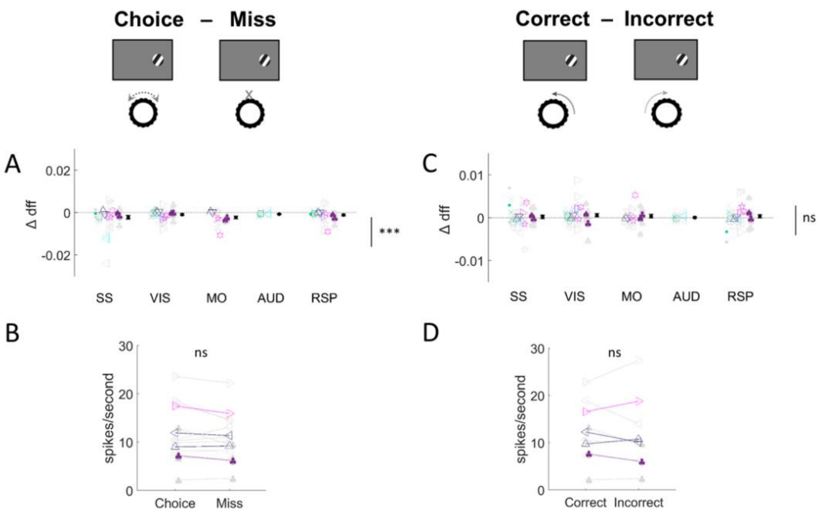

Figure S4. Differences in baseline activity do not explain the effects on 3-6Hz power. Related to Figure 3. A. Choice – Miss difference in dff fluorescence. There is a small but significant difference, with Miss baseline activity increased compared to Choice activity. The decrease in power can therefore not be driven by an increase in activity levels. **B.** Comparison of baseline firing rates from Neuropixels recordings. **C-D.** Similar analysis but comparing Correct and Incorrect trials.

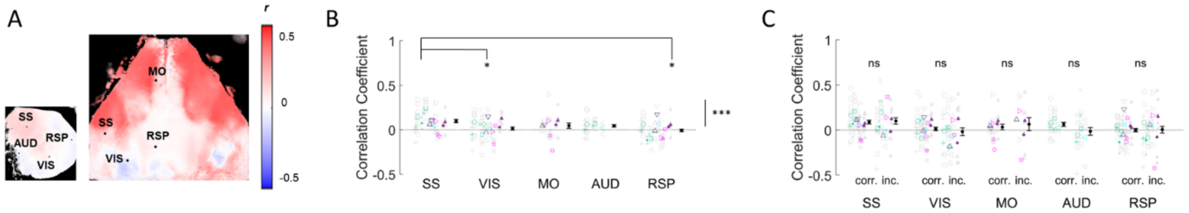

Figure S5. 3-6Hz power correlates with subsequent reaction time. Related to Figure 3. A. Pseudocolor maps showing correlation of reaction time with 3-6Hz power in each pixel. Red indicates a positive correlation: the lower the power, in other words the more desynchronized the cortical state, the faster the reaction time. **B.** Summary of power-reaction time correlations for all experiments. The correlation in somatosensory cortex was significantly stronger than in visual and retrosplenial cortex ($p<0.05$, one-way ANOVA. The significant overall effect was computed using one sample t-test for all correlations). **C.** Same as B but split into Correct (corr.) and Incorrect (inc.) Choice trials. There was no difference in correlation between 3-6Hz power and reaction time between Correct and Incorrect Choice trials in all ROIs, suggesting the correlation reflected a general readiness to respond and no selective effects depending on performance accuracy. *, $p < 0.05$; ***, $p < 0.001$; ns, $p \geq 0.05$

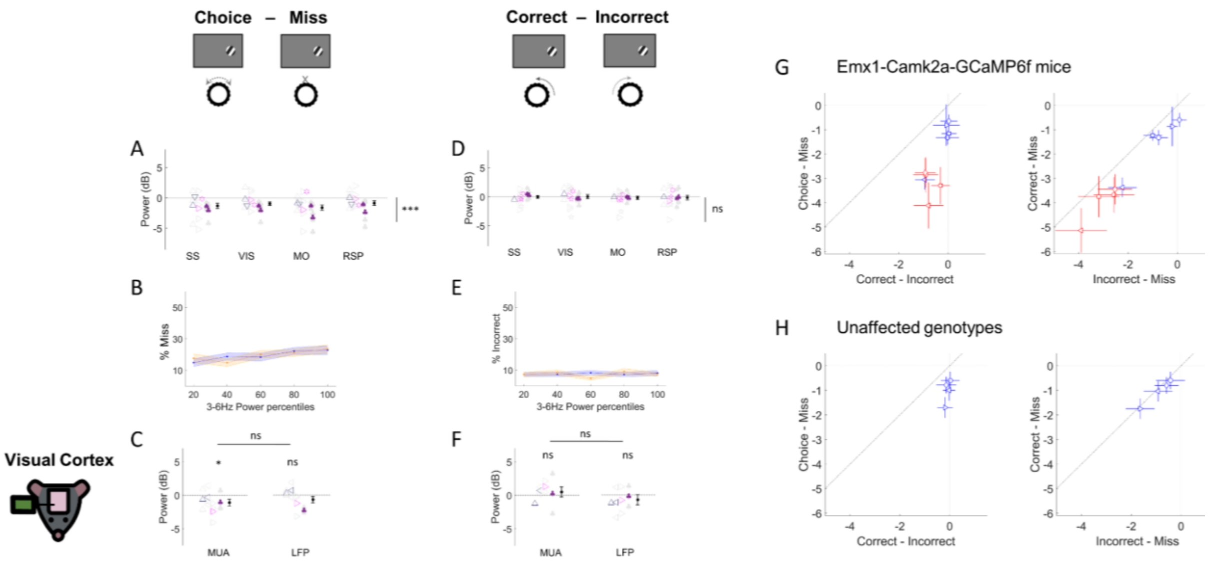

Figure S6. Excluding animals from genotypes that are prone to interictal activity did not change the results, and electrophysiological recordings in visual cortex replicated the same results as obtained by imaging. Related to Figures 3 and 7. A. Summary Choice-Miss 3-6Hz power differences ($p<0.001$, nested mixed ANOVA, n = 21 experiments from 6 animals excluding interictal-prone lines). Similar results were obtained when considering GCaMP6f animals alone (n = 38 experiments from 8 animals, $p<0.001$ nested mixed ANOVA) and GCaMP6s animals alone (n = 20 experiments from 7 animals, $p<0.001$ nested mixed ANOVA) separately. **B.** Percent Miss increases equally with increasing 3-6Hz power in visual (blue, $p<0.01$) and somatosensory (orange, $p<0.05$) cortex. **C.** 3-6Hz power differences in visual cortex as measured from multi-unit activity (MUA) and local field potentials (LFP) from Neuropixels recordings in some of the same animals that had previously been imaged and are shown in A. n = 10 experiments from 4 animals. **D-F.** Similar analysis as A-C but comparing Correct and Incorrect Choices. **E.** Visual cortex (blue): p=0.7, Somatosensory cortex (orange): p =0.9. **G-H.** Task related cortical state differences do not depend on genotype. **G.** Each point shows the power difference (dB) for Choice vs. Miss trials and Correct vs. Incorrect trials, averaged over all subjects, for a specific cortical area in in EMX1-Camk2a-GCaMP6f mice. Blue symbols are for visual task, red for auditory task. Error bars represent standard error of the mean over subjects. **H.** Results from mice of genotypes for which interictal activity has not been reported, in the visual task. *, $p<0.05$; ***, $p < 0.001$; ns, $p\geq0.05$

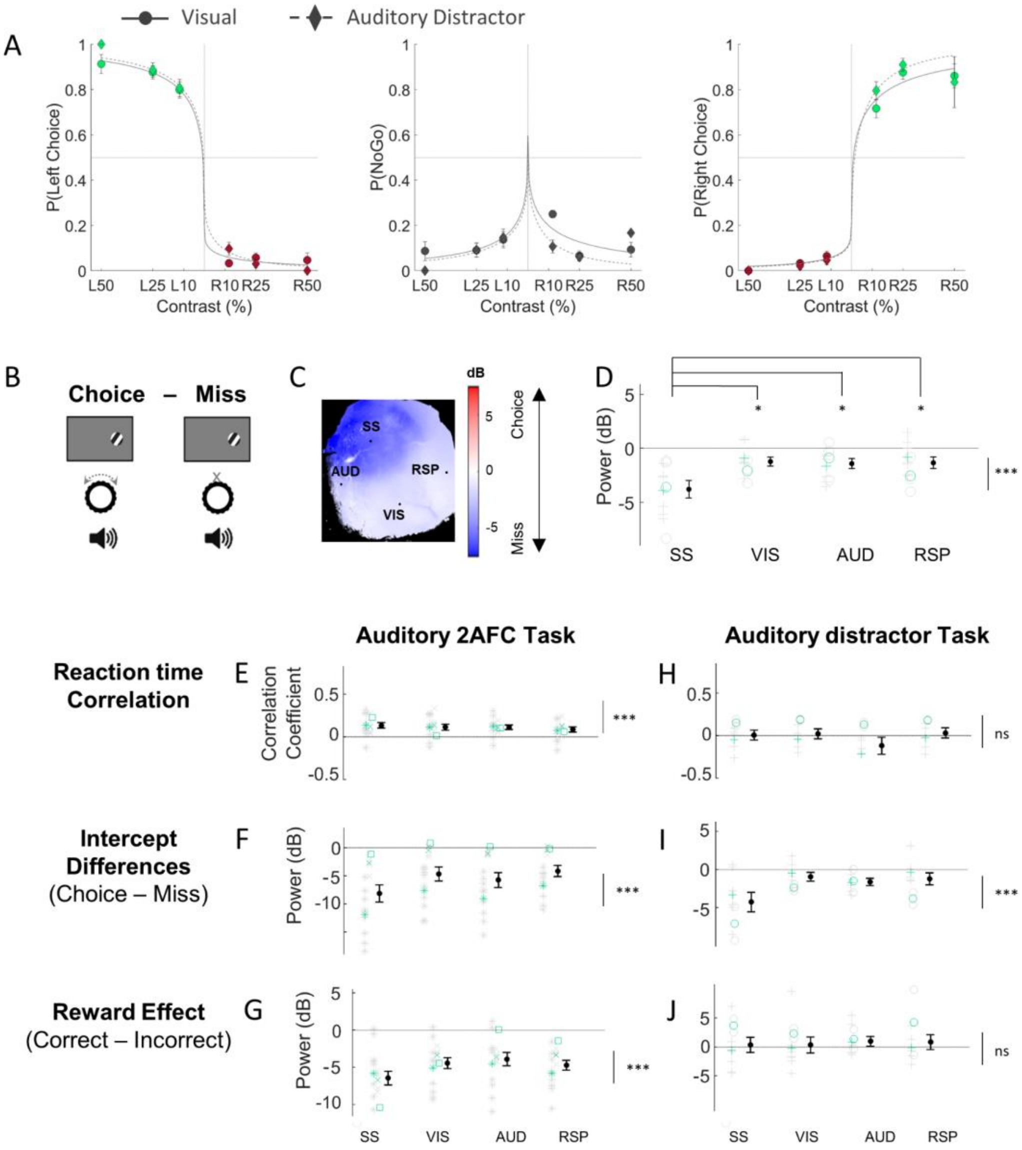

Figure S7. Results from the auditory 2AFC and auditory distractor tasks. Related to Figures 5, 6 and 7. A-D: The auditory distractor task. **A.** Psychometric curves comparing the performance during the normal visual and the auditory distractor task. The auditory distractor task consisted of the visual 2AFC task onto which irrelevant auditory tones were added (the same ones as in the auditory task, but they were inconsistently paired with the visual stimuli so as not to provide any extra information about the stimulus). The mice successfully disregarded the auditory stimuli and performed equally as well in the auditory distractor task as in the normal visual task. **B.** Cartoon illustrating the comparison of Choice and Miss trials in the auditory distractor task. **C.** Pseudocolor map showing the 3-6Hz power difference for each pixel; blue indicates higher power on Miss trials. **D.** Summary of 3-6Hz power differences between Choice and Miss trials for selected ROIs across all experiments in the auditory distractor task (n = 10 experiments from 2 animals). **E-J:** The results regarding reaction time correlation, relationship with pupil, and effect after reward are replicated in the auditory and auditory distractor tasks. *, $p < 0.05$; ***, $p < 0.001$; ns, $p \geq 0.05$
