# Supplementary material for: Cortical State Fluctuations during Sensory Decision Making
Source: Curr Biol. 2020 Dec 21;30(24):4944–4955.e7. doi: 10.1016/j.cub.2020.09.067 (PMC7758730; doi:10.1016/j.cub.2020.09.067)
Supplement: Document S2. Article plus Supplemental Information [file mmc2.pdf]

Article

Current Biology

Cortical State Fluctuations during Sensory Decision Making

Highlights

- Cortical 3- to 6-Hz fluctuations are more correlated with engagement than performance
- 3- to 6-Hz oscillations are global and do not depend on the sensory modality of the task
- Reduced slow oscillations may enable rapid, coordinated responses to sensory stimuli

Authors

Elina A.K. Jacobs,
Nicholas A. Steinmetz,
Andrew J. Peters, Matteo Carandini,
Kenneth D. Harris

Correspondence

elina.jacobs@ucl.ac.uk (E.A.K.J.),
kenneth.harris@ucl.ac.uk (K.D.H.)

In Brief

Jacobs et al. test the hypothesis that slow (3–6 Hz) cortical oscillations impair performance during sensory-guided decision-making tasks. They find that slow oscillations are more related to engagement than performance: mice are less likely to respond at all during increased slow oscillations, but no more likely to give an incorrect response.

Jacobs et al., 2020, Current Biology *30*, 4944–4955
December 21, 2020 © 2020 The Authors. Published by Elsevier Inc.
https://doi.org/10.1016/j.cub.2020.09.067

CellPress

CellPress
OPEN ACCESS

Current Biology

Article

Cortical State Fluctuations during Sensory Decision Making

Elina A.K. Jacobs,[1,3,*] Nicholas A. Steinmetz,[1,4] Andrew J. Peters,[2] Matteo Carandini,[2,5] and Kenneth D. Harris[1,5,6,*]
[1]UCL Queen Square Institute of Neurology, University College London, Queen Square, London WC1N 3BG, UK
[2]UCL Institute of Ophthalmology, University College London, Bath Street, London EC1V 9EL, UK
[3]Present address: UCL Sainsbury Wellcome Centre for Neural Circuits and Behaviour, University College London, Howland Street, London W1T 4JG, UK
[4]Present address: Department of Biological Structure, University of Washington, 1959 NE Pacific Street, Seattle, WA 98195, USA
[5]Senior author
[6]Lead Contact
*Correspondence: elina.jacobs@ucl.ac.uk (E.A.K.J.), kenneth.harris@ucl.ac.uk (K.D.H.)
https://doi.org/10.1016/j.cub.2020.09.067

SUMMARY

In many behavioral tasks, cortex enters a desynchronized state where low-frequency fluctuations in population activity are suppressed. The precise behavioral correlates of desynchronization and its global organization are unclear. One hypothesis holds that desynchronization enhances stimulus coding in the relevant sensory cortex. Another hypothesis holds that desynchronization reflects global arousal, such as task engagement. Here, we trained mice on tasks where task engagement could be distinguished from sensory accuracy. Using widefield calcium imaging, we found that performance-related desynchronization was global and correlated better with engagement than with accuracy. Consistent with this link between desynchronization and engagement, rewards had a long-lasting desynchronizing effect. To determine whether engagement-related state changes depended on the relevant sensory modality, we trained mice on visual and auditory tasks and found that in both cases desynchronization was global, including regions such as somatomotor cortex. We conclude that variations in low-frequency fluctuations are predominately global and related to task engagement.

INTRODUCTION

The cerebral cortex operates in multiple states, which can be distinguished by the strength of low-frequency fluctuations. Low-frequency fluctuations were seen in the earliest electroencephalogram (EEG) recordings [1], and their power is decreased by factors such as sensory stimuli, eye opening (including in the dark), or mental arithmetic [2–5], and increased by anesthesia or sleep [6, 7]. The scalp EEG and intracranial local field potential (LFP) are indirect measures of cortical spiking, reflecting synaptic currents arising from distal as well as local inputs, subthreshold activity, and electrical volume conduction from distant regions [8]. Nevertheless, it was hypothesized that low-frequency fluctuations represent a state where local neurons "beat in unison," occurring when a cortical area is "unoccupied," whereas engagement in sensory or non-sensory processing will "break up the synchronous beat" [4]. Decades later, simultaneous recordings of multiple neurons confirmed that increased amplitude of slow EEG/LFP fluctuations indeed correlates with increased synchrony of local neurons [2, 9–11].

Cortical states are continuous and not discrete: slow correlated fluctuations increase gradually through inalert waking, to a maximum in slow-wave sleep [12]. The synchronized state contains both irregular and rhythmic patterns at different frequencies, such as alpha (~10 Hz), delta (~4 Hz), and slow (~1 Hz) bands, depending on behavioral conditions. Here we use the term "synchronization" to refer to any ≤10-Hz fluctuations in local populations, excluding oscillations at higher (gamma) frequencies, which can increase in desynchronized states [13].

Although the general relationship between desynchronization and alertness is now firmly established, questions remain over its spatial structure. Desynchronization of the occipital alpha rhythm (~10 Hz) is strongest in visual cortex [4], but it is unclear whether different cortical regions desynchronize independently during specific tasks and whether such desynchronization improves sensory processing. Recordings in primates showed that low-frequency "noise correlations" (signatures of a more synchronized state) decrease with spatially selective attention, specifically in the region of visual cortex representing an attended part of the visual field [14–16]. Theoretical arguments suggest that correlated low-frequency fluctuations can impair information coding [17–20], and recordings in rodents suggest that cortical sensory representations are more reliable in desynchronized states [21–29]. This indicates that cortex might desynchronize in a localized manner, with the resulting decreased noise correlations improving representation of attended sensory stimuli.

 © 2020 The Authors. Published by Elsevier Inc.
This is an open access article under the CC BY license (http://creativecommons.org/licenses/by/4.0/).

A second, non-exclusive, hypothesis is that desynchronization is a consequence of a global change in brain state that covaries with performance on sensory tasks but need not causally contribute to it. Indeed, correlated fluctuations only impair sensory coding when their structure mimics that of sensory stimuli [17–20]. At least in mouse visual cortex, this is not the case: spontaneous fluctuations occur along dimensions largely orthogonal to visual responses [30, 31]. Instead, the poor behavioral performance that accompanies synchronized states might occur for reasons other than impaired cortical sensory representations, such as lack of motivation or engagement.

Here we investigated these questions by studying how cortical state in multiple regions correlates with behavioral performance. We used multialternative choice tasks, which allowed us to distinguish perceptual errors (choosing the wrong option) from differences in task engagement (failing to respond). We trained mice on tasks requiring different sensory modalities, and imaged population activity across dorsal cortex using widefield imaging of genetically encoded calcium indicators. We validated that decreased low-frequency power in the local widefield signal reflects local desynchronization using simultaneous electrophysiology. The results indicate that low-frequency fluctuations are predominately global and related to task engagement.

RESULTS

We trained mice in multiple decision-making tasks using visual and/or auditory modalities. We first present results from the purely visual task.

The Level of Task Engagement Varies throughout a Session

We trained 15 mice to perform several variants of a head-fixed visual decision-making task [32] (Figures 1A and 1B). In the two-alternative forced choice (2AFC) variant (5 mice), mice responded to Gabor stimuli appearing on the left or right by turning a steering wheel, and received a water reward for driving the stimulus to the center of the middle screen. Trials began after animals remained still for a “quiescent period” of 0.5–2.0 s (uniform distribution); a Go cue was played 0.3–0.8 s after visual stimulus onset, following which mice had 1.5–5.0 s to respond in order to receive a reward. Trials were classified as Correct Choice (turning the wheel as required to receive a reward), Incorrect Choice (turning the wheel in the wrong direction), and Miss (no response before the trial timed out). As described below, the primary difference in cortical states was observed between Miss trials and Choice trials (whether Correct or Incorrect). We therefore group Correct and Incorrect trials as “Choice” trials for many analyses. Additional task variants (10 mice; Figure 1B) included trials with no stimulus on either side, for which the animals were rewarded for refraining from turning the wheel [32, 33]. These yielded two additional trial types: Correct Rejects (providing a No-Go response by keeping the wheel still during zero-contrast trials) and False Alarms (turning in either direction during zero-contrast trials).

Task sessions lasted 20–60 min during which animals completed up to 400 trials and produced high-quality psychometric curves (Figures 1C–1E). Nevertheless, animals occasionally produced Miss responses (Figures 1D and 1F–1I). Miss trials were most common for low-contrast stimuli but could occur even at the highest contrasts. Misses often came in sequences: mice disengaged for several trials in a row, before re-engaging with the task ($p < 0.05$, t test comparing actual and shuffled Miss sequence lengths; Figures 1F–1I).

Engagement Correlates with Decreased Low-Frequency Power in Visual Cortex

To study the global structure of cortical state changes, we used widefield GCaMP imaging, which provided a robust readout of local spiking power up to ~8 Hz (Figures 2C and 2E). To show this, we performed widefield imaging simultaneously with Neuropixels [34] recordings in primary visual cortex (V1; Figures 2A–2F). We fit a linear spatiotemporal filter to predict multiunit spiking activity from the widefield calcium movie. Spiking was best predicted by local calcium activity (Figure 2A), with a sharp-rising/slow-decaying temporal kernel matching the fluorescence time course of GCaMP (Figure 2B). Fluorescence was coherent with local spiking and LFP signals up to ~8 Hz (Figures 2C and 2D), and power modulation was correlated over the same frequency range (Figures 2E and 2F). These correlations were not driven by changes in firing rate: they persisted when restricting analysis to periods of either high or low local spiking (Figure S1B). Spiking was more coherent with the local GCaMP signal than the LFP (coherence of 0.56 versus 0.31; $p < 0.05$, t test; Figures 2C, 2D, and S1A). To evaluate the relative contribution of local activity versus long-range axons to fluorescence, we applied the $GABA_A$ agonist muscimol to visual cortex, which abolishes only the former. Muscimol application decreased 3- to 6-Hz power to 18.7% ± 1.2% of the control condition (Figure S1D), indicating that up to 81% of the low-frequency widefield signal reflects local spiking.

V1 activity was more synchronized prior to Miss than Choice trials (Figures 2G–2M). We restricted our analysis of the cortical state during the task to the enforced quiescent period prior to stimulus onset (Figure 2G). This activity was therefore independent of the upcoming stimulus and excluded possible effects of ongoing wheel movement. We first focused our analysis on the region of primary visual cortex retinotopically aligned to the task stimuli (black dot in Figure 2H). We observed more low-frequency power in activity prior to Miss than Choice trials (Figures 2I–2M), with the largest differences around 6 Hz (Figure 2M). Because the brief duration of the quiescent periods did not allow analysis below 3 Hz, we focused on the 3- to 6-Hz band. Other recent studies in mouse visual cortex have also noted prominent, behaviorally modulated oscillations in this band [35, 36], which have been hypothesized to reflect a homolog of the human alpha rhythm [37].

Performance and low-frequency power showed a graded relationship. The fraction of Miss trials increased steadily with increasing 3- to 6-Hz power (Figure 2N; $p < 0.001$, generalized linear mixed-effects model). We obtained similar behavioral modulation of 3- to 6-Hz power by analyzing the spectrum of local population activity recorded with Neuropixels probes (Figures 2O and 2P), which also correlates well with measures of LFP power across multiple low-frequency bands (Figure S1C). Miss trials occurred at all contrast levels, and we observed differences in low-frequency power prior to Choice and Miss after restricting the analysis to either high- or low-contrast trials (Figures S2A and S2B).

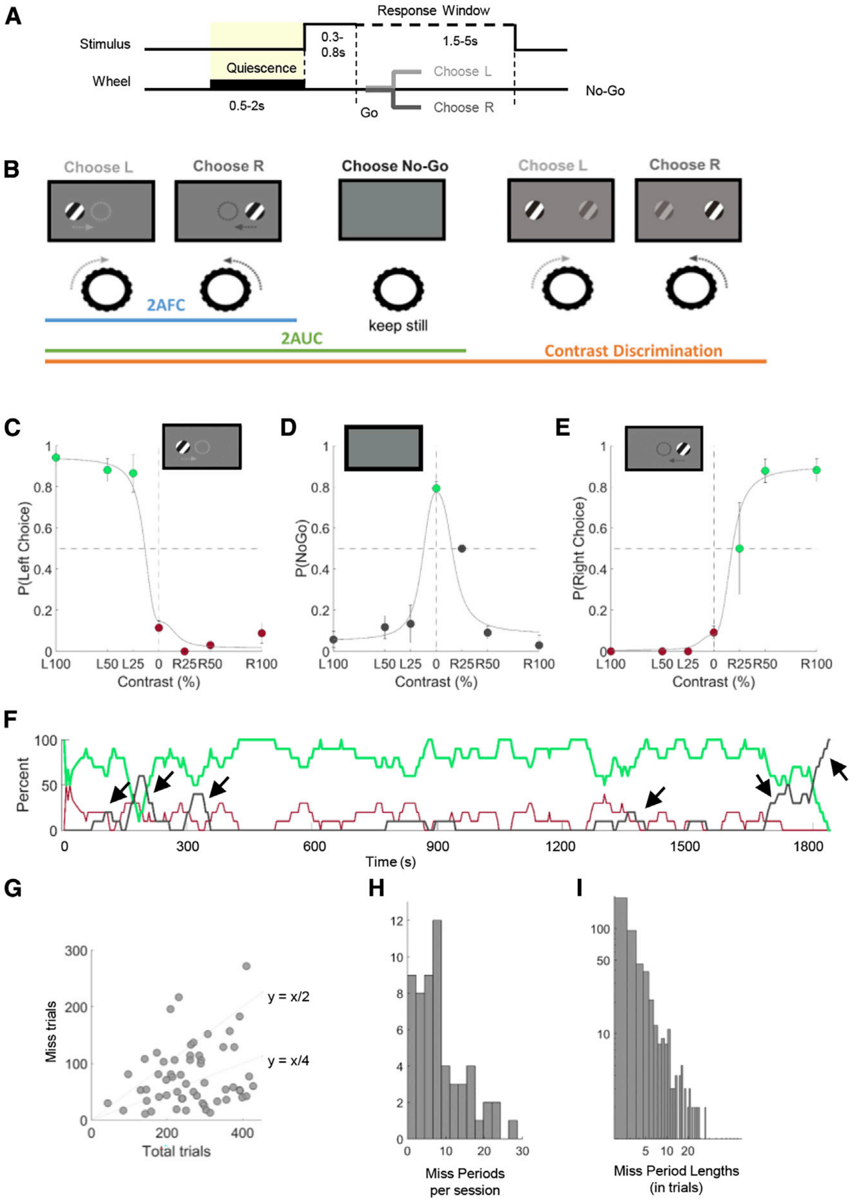

Figure 1. Level of Engagement Varies throughout a Session

(A) The trial structure of the task. A trial begins with a baseline period, which ends in a quiescent period (shaded in yellow) during which the mouse has to keep the wheel still. Then the visual stimulus appears, and a Go cue indicates the start of the response window, during which the mouse has to use the wheel to provide a response. The trial ends when the mouse provides a response or the response window ends. There is a feedback period before the beginning of the next trial.

(B) The visual tasks: in the 2AFC version, a Gabor grating of varying contrast appears on the left or right visual field. The mouse must move the wheel to center the stimulus and receive a reward. In the 2AUC version, trials are included in which the absence of a stimulus indicates a No-Go trial, during which the animals are rewarded for keeping the wheel still. In the contrast discrimination version, trials are included in which there is a contrast on both sides, and the stimulus with the higher contrast must be moved to the center. Equal contrast trials are rewarded randomly.

(C–E) Example psychometric curves. Error bars represent standard error of the mean (SEM). Dots are colored according to response type: green, Correct; red, Incorrect; dark gray, No-Go.

(F) Engagement over time in an example dataset that contained several short Miss sequences. Green, red, and dark gray curves show running average percentage of Correct, Incorrect, and Miss trials (10-trial running average). Arrows: Miss sequences.

(G) Total number of Miss trials per dataset versus total number of trials.

(H) Number of Miss periods (defined as 2 or more Miss trials in sequence) across all datasets.

(I) Miss period lengths across all datasets.

(A) and (B) were adapted with permission from [32].

Initial sensory responses encoded the visual stimuli equally well in Choice and Miss trials (Figures 2Q, S3B, and S3C). V1 fluorescence during the initial stimulus response (100–300 ms after stimulus onset) did not differ significantly between Choice and Miss trials or correlate with low-frequency power or other engagement variables (Figures S3C and S3E); at later time periods, stronger fluorescence was seen in Choice trials (Figure S3D), likely reflecting modulation by ongoing movement [31, 38]. The encoding of visual stimuli in the cortical population was also unaffected: we trained a decoder to predict contralateral stimulus presence from V1 population responses (50–150 ms after stimulus onset), and observed no difference in decodability between Choice and Miss trials (Figure 2Q; $p > 0.05$, nested mixed-effects ANOVA), low and high 3- to 6-Hz multiunit activity (MUA) power trials (Figure 2R; $p > 0.05$), or low and high 3- to 6-Hz LFP power trials (Figure 2U; $p > 0.05$). This unchanged decodability occurred despite a steady increase in population Fano factor (a measure of correlated spike count variability) with low-frequency power (Figure 2S; $p < 0.001$, generalized linear mixed-effects model) and there was no difference in decodability between low- and high-Fano-factor trials (Figure 2T; $p > 0.05$), suggesting that the variability associated with increased low-frequency power was not information limiting [18].

Performance-Related Power Differences Are Global and Correlate with Engagement More Than Perceptual Accuracy

We next asked whether the decrease in low-frequency power during Choice trials was specific to visual cortex, or global across cortex. To assess brain state across as many cortical regions as possible, we employed two different imaging strategies. For some animals, we imaged the entire dorsal cortical surface bilaterally, including visual, somatosensory, motor, and retrosplenial cortex; for others, we imaged the left cortical hemisphere unilaterally, to provide access to auditory cortex in addition to visual, posterior somatosensory, and retrosplenial cortex.

CellPress
OPEN ACCESS

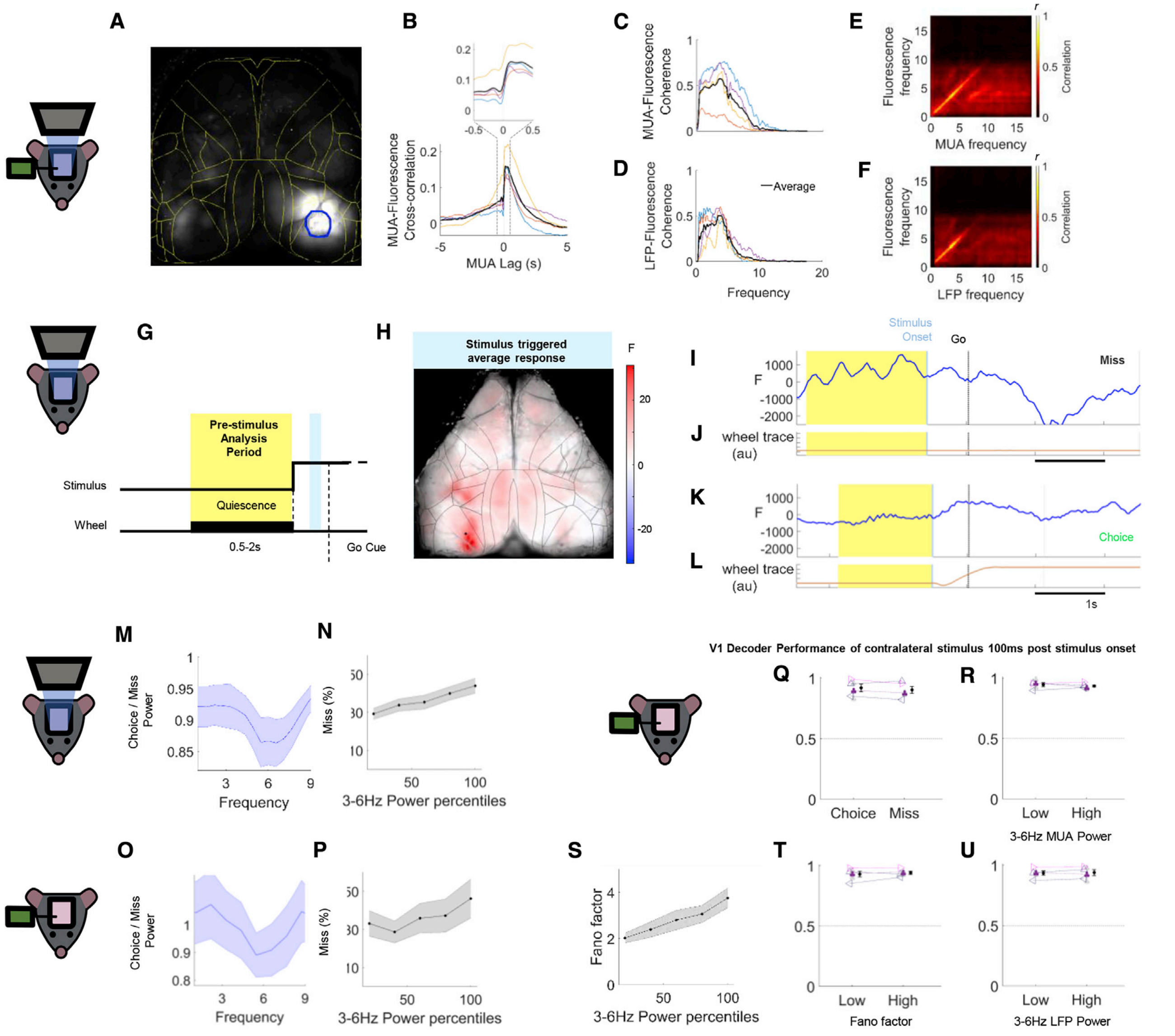

Figure 2. Engagement Correlates with Decreased Low-Frequency Power in Visual Cortex

(A) Pseudocolor representation of the regression weights optimally predicting spikes from fluorescence. Yellow lines: cortical area borders from the Allen atlas. Blue circle: Neuropixels probe location, from which the fluorescence signal used in (B)–(F) was obtained.

(B) Cross-correlation between multiunit activity (MUA) and fluorescence in visual cortex. Inset: zoom-in to the time period indicated by the dashed lines (bottom).

(C) Coherence between MUA activity and fluorescence. Each colored curve represents one mouse; the thick black curve indicates their average.

(D) Coherence of local field potential (LFP) and fluorescence.

(E) Average cross-frequency correlation between instantaneous MUA and fluorescence powers.

(F) Same as (E) but with LFP instead of MUA power.

(G) Schematic indicating pre-stimulus (quiescent period; yellow) and stimulus response (blue; 70–80 ms after stimulus onset) analysis periods. All trials started with a baseline of 1–5 s, during which animals had to remain quiescent for 0.5–2 s to initiate the appearance of a stimulus. After the stimulus appeared, a Go cue signaled the start of the response window. If the animals did not make a choice within the response window (1.5–5 s), a No-Go or Miss response was recorded.

(H) Pseudocolor representation of average fluorescence in a stimulus response window after presenting high-contrast visual stimuli in the right visual field. Black lines: cortical area borders from the Allen atlas. The black dot in the left hemisphere indicates a pixel chosen for analysis of visual cortical power.

(I–L) Single-trial calcium traces from representative Miss (I and J) and Correct Choice (K and L) trials. Yellow background indicates a quiescent period, during which there was no stimulus present and the animals held the wheel still. Light blue vertical line indicates stimulus onset; gray vertical dashed line indicates the Go cue (start of the response window). The brown traces below the blue fluorescence traces indicate the wheel movements.

(M) Ratio of visual cortical power spectra in Choice versus Miss trials in visual cortex, averaged over all experiments (n = 58 experiments from 15 animals; see STAR Methods). Shaded areas indicate SEM.

(N) Percent of Miss responses as a function of 3- to 6-Hz power.

(O and P) Same analysis as in (M) and (N) for MUA power in animals performing the same tasks with electrophysiology recordings (n = 11 experiments from 4 animals).

(legend continued on next page)

Performance-related desynchronization was neither specific to nor strongest in visual cortex (Figures 3A–3C). We observed a global decrease in 3- to 6-Hz power in Choice compared to Miss trials (Figures 3A and 3B; $p < 0.001$, nested mixed-effects ANOVA). Surprisingly, the largest effects were observed in somatomotor cortex (significant interaction between condition [Choice versus Miss] and region of interest [ROI], $p < 0.001$, nested mixed-effects ANOVA); the decrease in somatosensory cortex (SS) was larger than in retrosplenial (RSP; $p < 0.001$), visual, and auditory cortices (VIS and AUD, respectively; $p < 0.05$) but not different from secondary motor cortex (MO; $p > 0.05$, one-way ANOVA). Indeed, the percentage of Miss responses increased with 3- to 6-Hz power in both visual and somatosensory cortex (Figure 3C; $p < 0.001$, generalized linear mixed-effects model), with somatosensory cortex power having a significantly larger effect than visual cortex power ($p < 0.05$). The same results held if we excluded triple-transgenic animals [39] from the analysis (Figures S6A and S6B), if we considered only animals whose percent Correct at high contrasts exceeded 90%, or if we computed cortical state from different pre-stimulus time windows (results not shown). Similar results were also obtained using direct electrophysiological measurements of 3- to 6-Hz power in visual cortex (Figure S6C).

The global decrease in low-frequency power prior to Choice trials was not accompanied by an increase in either the mean wide-field signal or mean firing rates measured electrophysiologically (Figures S4A and S4B). Indeed, mean baseline fluorescence showed a small but significant decrease prior to Choice trials, consistent both with electrophysiological recordings in this task [38] and previous studies showing that desynchronized states do not increase mean rate unless accompanied by locomotion [28].

Pre-stimulus cortical state also correlated with reaction time. Choice trials with less pre-stimulus low-frequency power had faster reaction times; this correlation occurred globally (Figure S5; $p < 0.001$, one-sample t test for all correlations), with the strongest correlation in somatosensory cortex (SS versus RSP, $p < 0.001$; SS versus VIS, $p < 0.01$; SS versus MO and AUD, $p > 0.05$; one-way ANOVA).

Lastly, pre-stimulus cortical state showed a stronger correlation with task engagement than with perceptual accuracy (Figures 3E and 3F). To show this, we contrasted Incorrect Choice trials (where the subject engaged in the task but turned the wheel in the wrong direction, presumably reflecting sensory errors), with Miss trials (where the subject did not respond at all). Cortical state was comparable prior to Correct and Incorrect Choices: the difference was weak and only marginally significant ($p = 0.057$; Figure 3F), and the probability of wheel turns in the Correct versus Incorrect directions appeared unrelated to low-frequency power (Figure 3G; $p = 0.8$, generalized linear mixed-effects model). The difference between Choice and Miss trials was substantially larger than the difference between Correct and Incorrect Choices (Figure 3H). Furthermore, cortical activity was more desynchronized prior to Incorrect Choice than Miss trials ($p < 0.001$, nested mixed-effects ANOVA), with the difference between the two almost as large as between Correct Choice and Miss (Figure 3D). A similar effect was also seen in the 2AFC task variant, where the animals could not receive a reward for not turning the wheel, so Miss trials could not reflect an engaged subject that made a perceptual error ($p < 0.01$, nested mixed-effects ANOVA).

We therefore conclude that cortical desynchronization correlates with task engagement more than with perceptual accuracy: the main correlate of cortical state is not whether the subjects will choose correctly or incorrectly but whether they will respond at all, and furthermore how quickly they will respond to the stimulus.

Decreased 3- to 6-Hz Power Increases the Probability of Movement but Is Also Compatible with Movement Suppression

Previous work using a Go/No-Go paradigm showed that desynchronized states are associated not just with an increased tendency to respond to stimuli but also an increased tendency to produce False Alarm responses when a reward would have been obtained from withholding movement [27]. By using task variants that included elements of both left/right discrimination and Go/No-Go tasks (two-alternative unforced choice [2AUC] and contrast comparison; Figure 1B), we could also distinguish False Alarm responses from perceptual errors.

Animals were more likely to respond in desynchronized states, even when no visual stimulus was present. On zero-contrast trials, cortical activity prior to False Alarm trials was more desynchronized than prior to Correct Reject trials (Figures 4A and 4B; $p < 0.001$, nested mixed-effects ANOVA), and furthermore the probability of a False Alarm increased with pre-stimulus desynchronization (Figure 4C; VIS: $p < 0.01$; SS: $p = 0.1$; generalized linear mixed-effects model). Nevertheless, animals were still able to withhold movements in desynchronized states. When comparing two trial types in which no movement occurred, Correct Reject versus Miss, the cortex was more desynchronized on Correct Reject than on Miss trials (Figures 4D and 4E; $p < 0.001$, nested mixed-effects ANOVA), consistent with a higher state of engagement even though no movements occurred. Thus, increased desynchronization is associated with a propensity toward movement, but animals are still able to correctly withhold movements in desynchronized states in order to obtain rewards.

Cortical States Are Not Fully Explained by a General Measure of Arousal as Inferred from Pupil Fluctuations

Pupil fluctuations correlate with arousal and mental effort in humans, and with cortical state in rodents [25, 27, 40, 41]. We therefore asked how pupil measures related to engagement

(Q and R) Performance of a logistic regression decoder trained on primary visual cortical population activity to detect the presence of a contralateral stimulus. There were no significant differences in decoder performance between Choice and Miss trials (Q), or low and high 3- to 6-Hz power in MUA (R) trials ($p > 0.05$; $n = 5$ experiments from 4 animals). Color indicates genotype; different glyphs indicate different animals. For one of the animals there were two sessions; the individual sessions are indicated in light gray; the colored glyph indicates their average. Dashed lines connecting two glyphs indicate paired experiments; the dashed line at 0.5 indicates chance-level decoding.

(S) Noise correlations, as inferred from the Fano factor of summed population activity, increase with increasing 3- to 6-Hz MUA power.

(T and U) There was no significant difference in decoder performance between trials with low or high Fano factor (T) or low or high 3- to 6-Hz LFP power (U) ($p > 0.05$).

See also Figures S1–S3.

CellPress
OPEN ACCESS

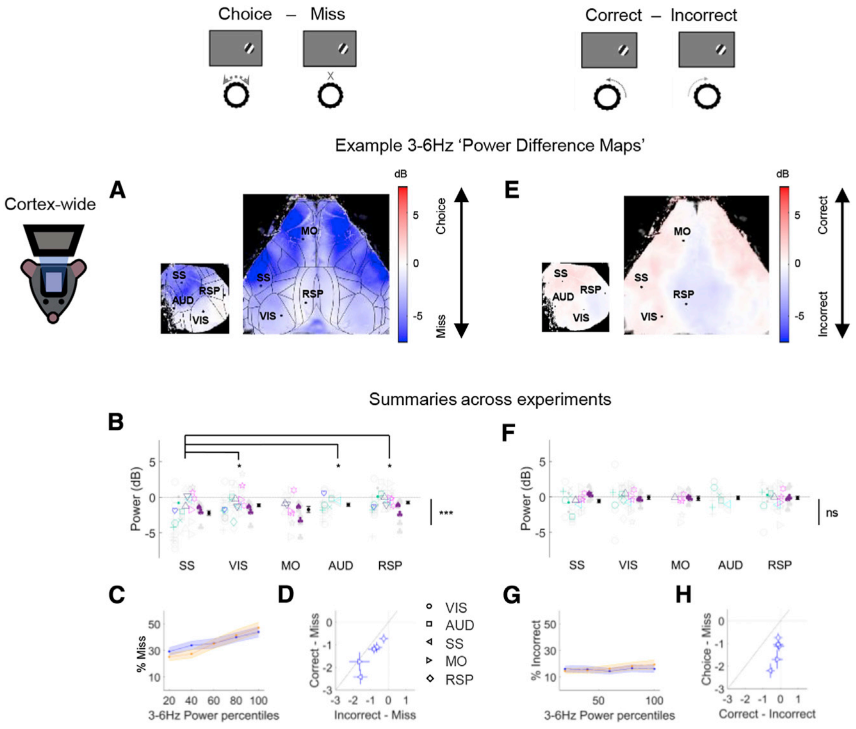

Figure 3. Performance-Related Power Differences Are Global and Correlate with Engagement More Than Perceptual Accuracy

Top: cartoons illustrating the trial types being compared.

(A) Example maps showing the difference in 3- to 6-Hz power between Choice and Miss trials, in pseudocolor for each pixel. Blue indicates greater power in Miss trials. Left: unilateral imaging. Right: bilateral imaging (same dataset as previous 2 figures). Black lines: Allen atlas cortical boundaries. Dots indicate pixels used as regions of interest (ROIs: primary visual cortex, VIS; primary somatosensory cortex, SS; primary auditory cortex, AUD; retrosplenial cortex, RSP; secondary motor cortex, MO).

(B) Summary of 3- to 6-Hz power difference between Choice and Miss trials for selected ROIs across all experiments (n = 58 experiments from 15 animals). Negative values indicate stronger low-frequency power preceding Miss trials. Color indicates genotype; different glyphs indicate different animals (see also Animals in STAR Methods). Individual datasets are shown in gray; the average per animal is shown in color. The average with SEM across all experiments is shown in black. Main effect of behavioral condition (Choice versus Miss, Correct Choice versus Incorrect Choice) is illustrated by the bar to the right of the summary graphs and was tested using a nested mixed-effects ANOVA. Post hoc tests to assess differences between areas used one-way ANOVAs.

(C) Percent Miss as a function of 3- to 6-Hz power in visual cortex (blue) and somatosensory cortex (orange).

(D) Both Incorrect and Correct Choice trials are more desynchronized than Miss trials. Each point represents the average across sessions for one brain region (symbol in legend); error bars represent SEM. x and y axis units: power difference (dB).

(E–G) Similar analysis comparing Correct and Incorrect Choice trials. No significant relationship with power was found.

(H) The cortical state difference between Choice and Miss trials is larger than the difference between Correct and Incorrect. Conventions are as in (D).

*$p < 0.05$, ***$p < 0.001$; ns, $p \geq 0.05$ (not significant).

See also Figures S4–S6.

and cortical state, and whether the correlation between engagement and cortical state could be explained by a common effect of arousal as measured by pupil changes.

Pupil size correlated with behavior and low-frequency power. Pupil size correlated negatively with probability of Miss trials (Figure 5A; % Miss: $p < 0.01$, generalized linear mixed-effects model) and low-frequency power during the quiescent period: the smaller the pupil, the greater the low-frequency power (Figures 5B and 5D). Nevertheless, pupil size did not fully explain the state-engagement correlation: even after accounting for pupil size, low-frequency power was stronger in Miss trials (Figures 5B–5D; $p < 0.05$, ANCOVA [analysis of covariance]). A consistent main effect of behavioral condition on low-frequency power was present in all ROIs after accounting for pupil size (Figures 5E and 5F; $p < 0.001$, nested mixed-effects ANOVA). The temporal derivative of pupil size also did not fully explain the state-engagement correlation (Figure 5G; $p < 0.001$, nested mixed-effects ANOVA). Thus, task engagement is not solely dependent on externally visible arousal as indicated by the pupil but also involves internal cognitive variables.

Reward Is Followed by a Prolonged Decrease in 3- to 6-Hz Power

Cortex desynchronized globally following rewards, and this desynchronization was unrelated to wheel turns and outlasted the act of reward consumption. As described above, low-frequency power differed at most weakly between the quiescent periods prior to Correct or Incorrect Choices (Figures 3E and 3F). However, cortical state did differ significantly in the quiescent periods that came after Correct and Incorrect Choices. To show this, we restricted our analysis to the quiescent period of the following trial (Figure 6A), by which time the animals were no longer moving the steering wheel and had finished licking (as measured by a thin-film piezo sensor attached to the lick spout) in 98% of trials (6,148/6,250). 3- to 6-Hz power in this quiescent period was lower following Correct compared to Incorrect Choice trials (Figures 6B–6D; $p < 0.001$, nested mixed-effects ANOVA). To exclude the possibility that increased visual contrast in rewarded trials could be driving the difference, we repeated the analysis separately for low and high contrasts, and the results persisted (Figures S2E and S2F). Similarly, power was lower following Correct Reject trials (no visual stimulus and no turn followed by reward) than following Incorrect Choice trials (visual stimulus and wrong-direction turn followed by no reward) (Figures 6E–6G; $p < 0.001$, mixed random effects ANOVA); again desynchronization followed rewarded trials, even though here the reward was earned by not moving. Thus, reward has a desynchronizing effect on brain state that persists several seconds after reward consumption has finished.

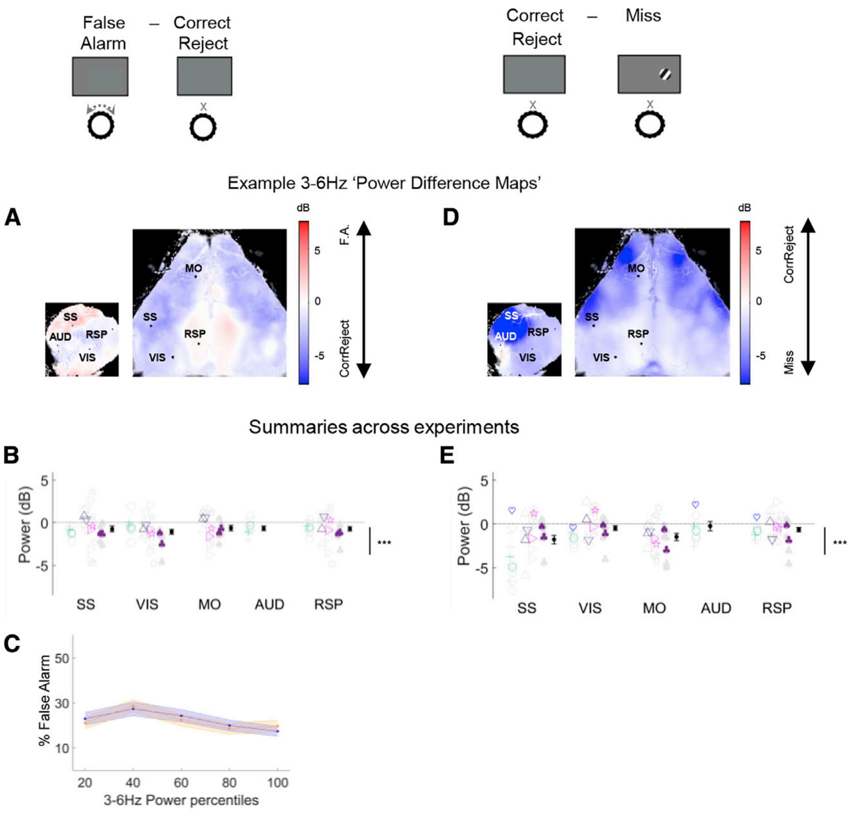

Figure 4. Decreased 3- to 6-Hz Power Increases Propensity to Move but Also Allows Movement Suppression

Top: cartoons illustrating the trial types being compared.

(A) Comparison of False Alarm (wheel turns during zero-contrast trials, which do not earn a reward) versus Correct Reject trials (where a reward is earned by refraining from turning). Example maps showing 3- to 6-Hz power difference; blue indicates higher power in Correct Reject trials.

(B) Summary of 3- to 6-Hz power difference during the quiescent period across experiments.

(C) Percent False Alarm as a function of 3- to 6-Hz power in visual (blue) and somatosensory (orange) cortex.

(D and E) Same analysis for comparison of Correct Reject versus Miss trials.

***$p < 0.001$.

Engagement-Related Global Decrease in Low-Frequency Power Is Independent of Sensory Modality

Our results showed that desynchronization related to performance in a visual task was global, rather than restricted to visual cortex. We next asked whether performing an auditory task would also cause global desynchronization. In this task, the animals observed a uniform gray screen (as in the visual task), but no visual stimuli were present. Instead, they were presented with trains of high- or low-frequency auditory tones. Turning the steering wheel changed the sound frequency of the tone trains, and the mice were rewarded for bringing the stimulus frequency to a central target tone (Figures 7A and 7B).

In the auditory task, desynchronization was again seen over the entire imaged window when comparing Choice to Miss trials (Figures 7C–7E; $p < 0.001$, nested mixed-effects ANOVA). As in the visual task, Incorrect Choice trials were globally more desynchronized than Miss trials ($p < 0.001$, nested mixed-effects ANOVA). Here, the weak difference in global power between Correct and Incorrect Choice trials did reach statistical significance (Figures 7F–7H; $p < 0.001$, nested mixed-effects ANOVA), although the difference between Correct and Incorrect Choices was still substantially smaller than between Choice and Miss trials (Figure S6G), suggesting again that desynchronization related better to behavioral engagement than to perceptual accuracy. Also similar to the visual tasks, low-frequency power was significantly correlated with reaction time across the cortex (Figure S7E).

We also trained a subset of mice in an auditory distractor task (Figures S7A and S7B), in which mice were presented with both visual and auditory tone train stimuli that changed in tonal frequency as the wheel was turned, but the contingency of the auditory stimulus changed between blocks. Mice showed similar performance in this task as in the purely visual task (Figure S7A), suggesting they disregarded the auditory stimuli. In the auditory distractor task, brain-state changes closely mirrored those in the visual and auditory tasks: we saw a global decrease in low-frequency power during task engagement (Figures S7B–S7D; $p < 0.001$, nested mixed-effects ANOVA). Desynchronization was strongest in somatosensory cortex ($p < 0.01$; SS versus VIS, AUD, and RSP: $p < 0.05$, one-way ANOVA), but there were not sufficient Incorrect Choice trials to analyze differences between Correct and Incorrect, or Incorrect and Miss trials. The correlation of low-frequency power with reaction time did not reach significance (Figure S7H), but we observed the same relationship with pupil size in the auditory and auditory distractor tasks (Figures S7F and S7I) and found the same effect of reward in the auditory task, although this did not reach significance for the auditory distractor task (Figures S7G and S7J).

DISCUSSION

We trained mice on several discrimination tasks using visual and auditory sensory modalities. We assessed cortical states by spectral analysis of widefield GCaMP imaging, and confirmed with simultaneous electrophysiological recordings that it could reveal low-frequency fluctuations in local population spiking. In all tasks, we found that fluctuations in engagement throughout a session correlated with cortical state. Consistent with many prior studies [24, 27, 42], we found that responses to visual or auditory stimuli were more likely in states with decreased low-frequency power. However, we made two new discoveries. First, this decrease was observed in all recorded areas rather than solely in the sensory region corresponding to the stimulus modality. Second, the primary behavioral correlate was whether mice responded to a stimulus, and not whether they responded correctly.

CellPress
OPEN ACCESS

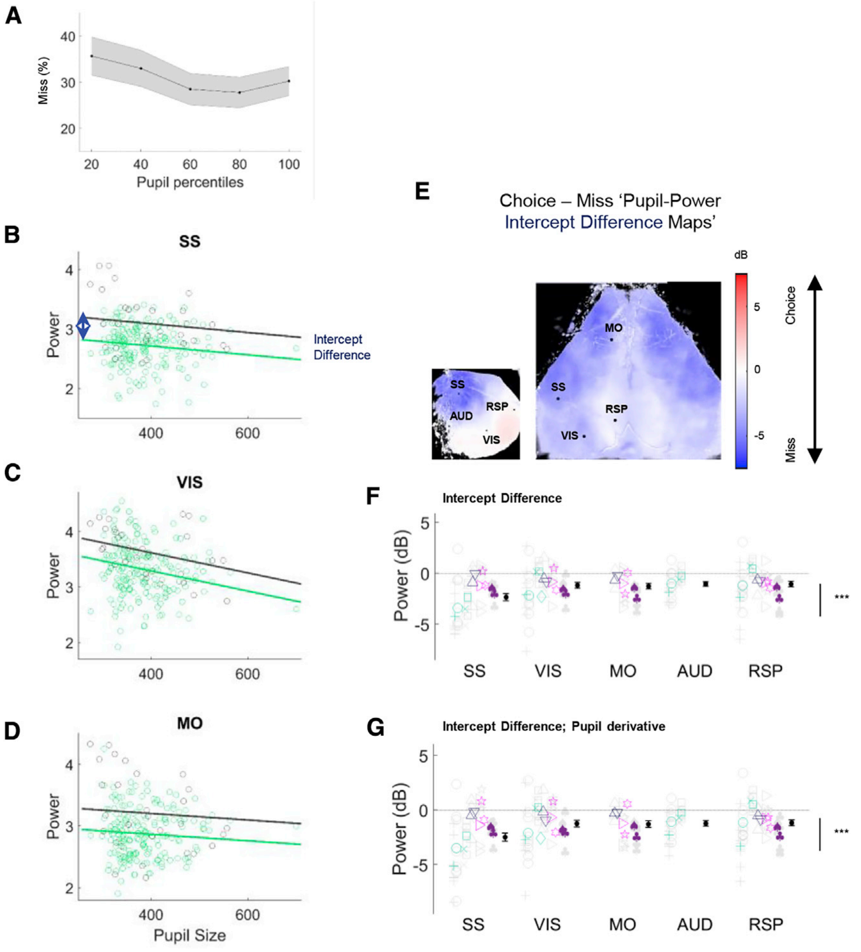

Figure 5. Variations in Cortical State Are Not Fully Explained by Variations in Pupil Size

(A) Probability of Miss trials at increasing pupil sizes.

(B) Relationship between pupil size, behavioral condition, and 3- to 6-Hz power in somatosensory cortex of an example experiment. Each dot represents a trial, colored according to outcome: Choice (green) or Miss (dark gray). The lines represent fits from an ANCOVA model, which captured the main effect of behavioral condition as the difference in intercept between the Choice and Miss fits (blue arrow).

(C and D) Similar analysis for visual and motor cortex ROIs.

(E) Pseudocolor maps showing intercept difference for each pixel, for the same example sessions shown in previous figures. Blue indicates significantly higher power prior to Miss trials, after accounting for the common effect of pupil size.

(F) Summary of intercept differences across experiments in selected ROIs (n = 48 experiments from 12 animals).

(G) Same analysis as in (F) but with the temporal derivative of pupil size.

See also Figure S7.

Our analyses focused on the 3- to 6-Hz band as a measure of low-frequency power. The prominence of this band in rodent cortex has been noted in previous studies, and it has been hypothesized as a rodent homolog of the primate alpha rhythm [35–37]. Power in this band was correlated with other low-frequency bands as assessed by widefield calcium, LFP, and spiking activity (Figure S1C), as well as correlated spike count variability. However, our study does not rule out a correlation of fluctuations in other frequency ranges with performance. For example, gamma oscillations (too fast to be seen with calcium imaging) have been implicated in the control of sensory processing [13, 43–47], whereas infra-slow oscillations (<1 Hz; too slow to be measured in our time windows) can modulate fluctuations in several frequency bands, including the ones we investigated [48]. Future experiments employing faster techniques such as voltage imaging [30, 49–52] in tasks such as ours that disambiguate between different types of errors could address how the global structure of these different frequency bands relates to task performance.

Behavior-Related State Changes Are Global

Most studies investigating the correlation between behavior and cortical state have focused on single cortical regions, associated with the sensory modality required for the task. Unexpectedly, we found that the strongest correlations with performance in a visual task in mice were not local to visual cortex but global, and indeed were strongest in somatomotor cortex. This seems to contradict work in primate visual cortex where, during spatial attention tasks, reductions in correlated fluctuations occur locally in parts of visual cortex corresponding to attended locations [14–16, 44, 53–55]. Similarly, a recent study showed that mice trained in a hemisphere-selective task could exhibit a unilateral modulation of visual cortical state [56]. We suggest the following, non-exclusive possible explanations for this difference. First, whereas these studies compared different subregions of a single cortical area, our study compared cortical areas corresponding to different sensory modalities. Second, it is possible that differences in task demands between these experiments led to different strategies for solving them, and that our task can be solved without local desynchronization. For example, local desynchronization might only occur when selective attention must be deployed to a particular part of the visual world, which was not the case in our experiments. Lastly, the effects observed in our experiments might reflect arousal and engagement, which may activate more global state mechanisms, rather than selective attention, which may have more local effects.

Peculiarly, the region in somatomotor cortex where we saw the strongest effect corresponded stereotaxically to barrel cortex, which was unanticipated given that there was no overt need to use the whisker system in any of our tasks. We found no difference in overall whisker motion between engaged (Choice) and disengaged (Miss) trials (results not shown). Nevertheless, we cannot exclude differences in whisker movements too subtle to be picked up with our measurement tools. Moreover, although the bodies of the mice were restrained, they

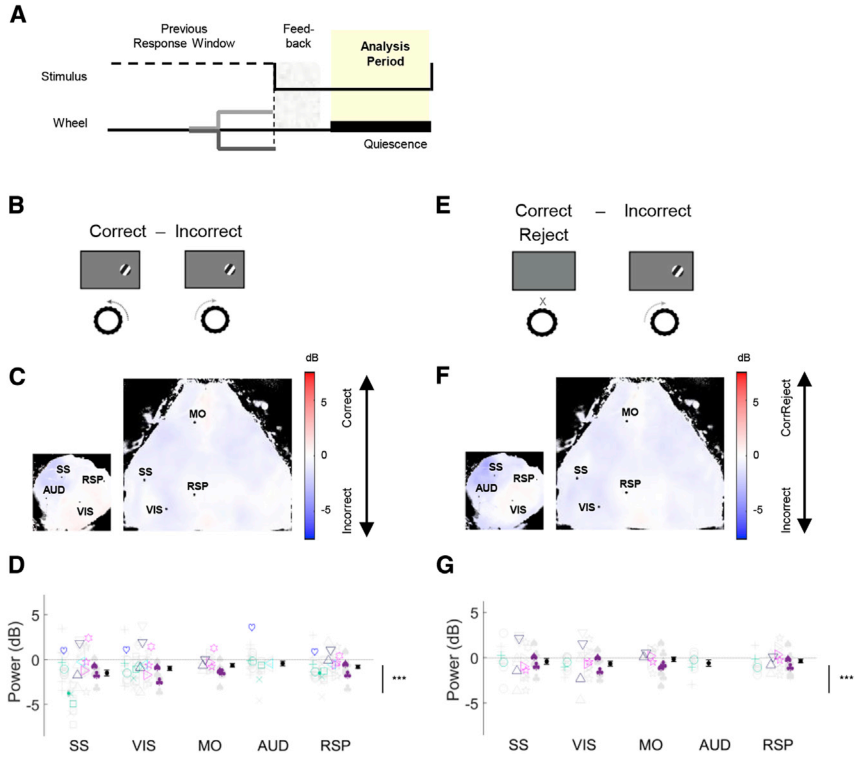

Figure 6. Reward Is Followed by Prolonged Decrease in 3- to 6-Hz Power

(A) Illustration of the analysis of quiescent periods following Correct and Incorrect Choice trials.

(B) Cartoon illustrating comparison of Correct and Incorrect Choice trials.

(C) Pseudocolor representation of 3- to 6-Hz power difference for each pixel; blue represents lower power following Correct Choice.

(D) Summary across experiments for selected ROIs (n = 57 experiments from 14 animals).

(E–G) Similar analysis for the comparison of Correct Reject and Incorrect Choice trials (n = 30 experiments from 9 animals).

See also Figures S2 and S7.

may have made small movements that we could not monitor, leading to somatomotor desynchronization. Lastly, it is possible that the desynchronization in somatomotor cortex reflects a state of readiness to provide a response in order to obtain a reward. This may be independent of the sensory processing, which could be occurring in parallel to a motor preparation.

Desynchronization Reflects a Behavioral State that Is Primed for Action

Desynchronized states are characterized by lower noise correlations, and it has been hypothesized that the resulting improvement in representation of sensory stimuli underlies improved performance in sensory discrimination tasks [14, 15, 20]. However, we did not find a strong correlation between desynchronization and increased behavioral accuracy, or with better decoding of stimuli from cortical activity, which this hypothesis would predict for our task. The most parsimonious explanation of our data is that desynchronization correlates primarily or solely with behavioral engagement, and that animals are almost as engaged during Incorrect as during Correct trials, despite sensory errors. The difference in cortical state between Incorrect and Correct trials was small, but did reach significance for the auditory task. It is possible that the auditory task, which was more difficult for the mice (as judged by the percentage successfully trained and the time taken to train them), required stronger engagement than the visual task. Thus, although our data cannot rule out that corruption of sensory representations by synchronized state fluctuations contributes to the state-behavior correlations we observed, this contribution is most likely substantially smaller than the contribution of task engagement.

Previous work using an auditory Go/No-Go task showed an “inverted U” curve of performance with cortical state, optimal in intermediate states of moderate desynchronization [27]. McGinley et al. showed that the types of error found in the two extremes were different: false negatives in the most synchronized states, and false positives in the most desynchronized [27]. Similarly, we found that Miss trials (when a stimulus was present but the mice failed to turn the wheel) increased linearly with low-frequency power, whereas False Alarm trials (when no stimulus was present but the mouse still turned the wheel) decreased with low-frequency power. Our results are thus consistent with their model, and we further provide evidence that neither type of mistake is due to perceptual error but rather to behavioral readiness.

Additionally confirming the interpretation that desynchronization indicates behavioral readiness, Miss responses occurred least at large pupil sizes, associated with behavioral arousal [25, 27]. Reaction times were faster in desynchronized states, regardless of Correct and Incorrect Choices, further suggesting that desynchronization is a state that prepares the animal for rapid and coordinated responses to sensory stimuli.

Finally, rewards also led to increased desynchronization. Rewards signal that the action taken led to a desirable outcome, which is likely to increase motivation, and therefore increase arousal and engagement. In addition to the well-established role of dopamine in action initiation and reward processing [57, 58], dopamine is also known to modulate cortical states [59, 60]. Although dopaminergic projections to visual cortex are weak, the effect we observed could be an indirect result of dopaminergic signals, through projections to cholinergic and noradrenergic nuclei, or top-down modulation from frontal areas that receive dopaminergic inputs [61–63] and carry reward signals [64].

Conclusions

Our results suggest that cortical desynchronization is not required for accurate sensory processing in this task but rather reflects a state that is associated with producing rapid and coordinated behavioral responses to sensory movements of any modality. The movements the mouse must make in this task involve large regions of the body; making them rapidly may require

CellPress
OPEN ACCESS

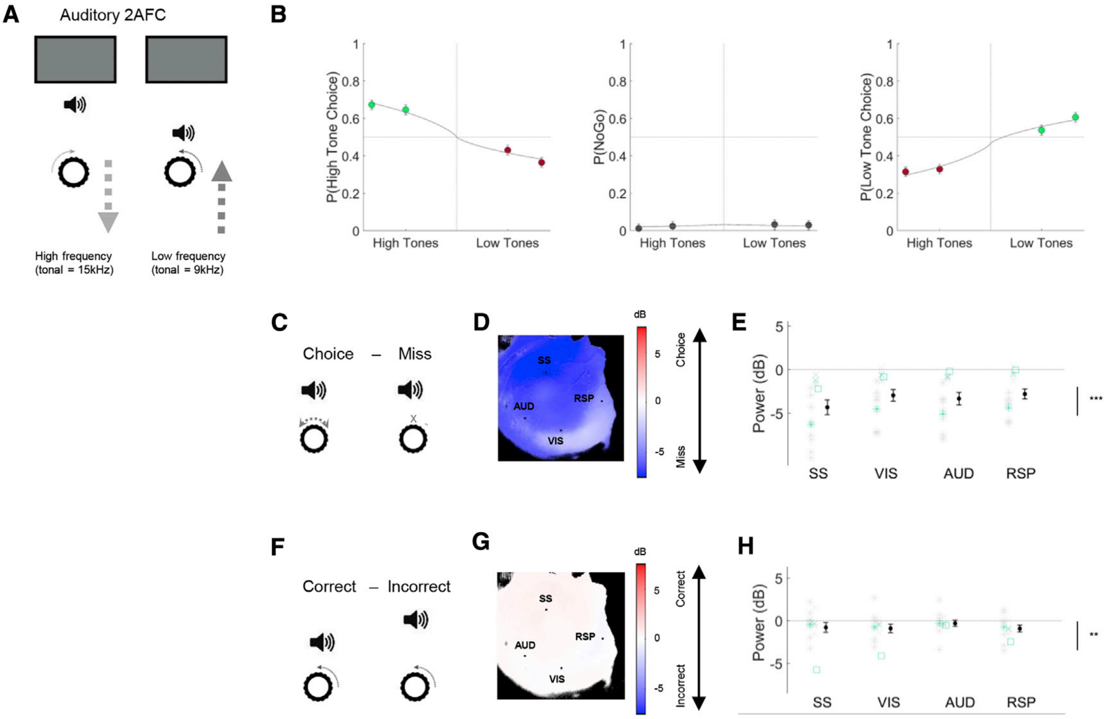

Figure 7. Engagement-Related Decrease in Low-Frequency Power Is Independent of Sensory Modality

(A) Illustration of the auditory 2AFC task. Mice were positioned in front of the same screen as during the visual tasks but it remained isoluminant without any stimuli. A speaker was placed at the bottom of the screen, through which low- and high- (tonal) frequency trains were heard. Turning the wheel modulated the (tonal) frequency of the stimuli, and the mice had to bring the stimulus to a central frequency.

(B) Average psychometric curve from the auditory task. The difficulty of the trials was manipulated by using different sound amplitudes (x axis). Dots are colored according to response type: green, Correct; red, Incorrect; dark gray, Miss.

(C) Comparison of Choice and Miss trials in the auditory task.

(D) Pseudocolor map showing 3- to 6-Hz power difference for each pixel; blue indicates higher power on Miss trials.

(E) Summary of 3- to 6-Hz power difference between Choice and Miss trials for selected ROIs across all experiments in the auditory task (n = 15 experiments from 3 animals).

(F–H) Comparison of Correct Choice versus Incorrect Choice trials in the auditory 2AFC task.

See also Figures S6 and S7.

widespread desynchronization of the entire cortical surface, particularly somatomotor cortex.

STAR★METHODS

Detailed methods are provided in the online version of this paper and include the following:

- KEY RESOURCES TABLE
- RESOURCE AVAILABILITY
  - Lead contact
  - Materials availability
  - Data and Code availability
- EXPERIMENTAL MODEL AND SUBJECT DETAILS
  - Animals
  - Surgery
- METHOD DETAILS
  - Behavioral tasks
  - Widefield imaging
  - Dimensionality reduction
  - Hemodynamic correction
  - Electrophysiological recordings
  - Eye tracking
  - Behavioral measurements
- QUANTIFICATION AND STATISTICAL ANALYSIS
  - Stimulus-triggered responses
  - Power maps
  - ROI selection
  - MUA and LFP power
  - Fano factor
  - Decoder analysis
  - Power percentiles
  - Statistics

SUPPLEMENTAL INFORMATION

Supplemental Information can be found online at https://doi.org/10.1016/j.cub.2020.09.067.

ACKNOWLEDGMENTS

We thank Michael Okun and Peter Zatka-Haas for contributing code for the data analysis, Philip Coen, Laura Funnell, and Miles Wells for help with animal

training, and Charu Reddy for animal husbandry. This work was supported by a Wellcome Trust PhD Studentship (to E.A.K.J.) (103364/Z/13/Z), a Wellcome Trust Investigator Award (205093/Z/16/Z), grants from the European Research Council (694401) and Simons Foundation (325512), and postdoctoral fellowships from the Human Frontier Sciences Program (to N.A.S. and A.J.P.) (LT001071/2015-L and LT226/2016-L), the European Union's Horizon 2020 research and innovation programme under Marie Skłodowska-Curie grant agreement 656528 (to N.A.S.), Royal Society Newton International Program (to A.J.P.) (NF150554), and EMBO (ALTF 1428-2015). M.C. holds the GlaxoSmithKline/Fight for Sight Chair in Visual Neuroscience.

AUTHOR CONTRIBUTIONS

Conceptualization, E.A.K.J., M.C., and K.D.H.; Methodology, E.A.K.J., N.A.S., A.J.P., M.C., and K.D.H.; Investigation, E.A.K.J., A.J.P., and N.A.S.; Software, E.A.K.J., N.A.S., A.J.P., and K.D.H.; Analysis, E.A.K.J. and K.D.H.; Writing – Original Draft, E.A.K.J. and K.D.H.; Writing – Review & Editing, N.A.S., A.J.P., and M.C.; Visualization, E.A.K.J. and K.D.H.; Supervision, M.C. and K.D.H.

DECLARATION OF INTERESTS

The authors declare no competing interests.

Received: December 23, 2019
Revised: July 28, 2020
Accepted: September 21, 2020
Published: October 22, 2020

REFERENCES

1. Caton, R. (1875). The electric currents of the brain. BMJ *2*, 278.
2. Coenen, A., Zajachkivsky, O., and Bilski, R. (1998). In the footsteps of Beck: the desynchronization of the electroencephalogram. Electroencephalogr. Clin. Neurophysiol. *106*, 330–335.
3. Berger, H. (1929). Ueber das Elektrenkephalogramm des Menschen. Arch. Psychiatr. Nervenkr. *87*, 527–570.
4. Adrian, E.D., and Matthews, B.H.C. (1934). The Berger rhythm: potential changes from the occipital lobes in man. Brain *57*, 355–385.
5. Beck, A. (1891). Oznaczenie lokalizacyi w mozgu i rdzeniu za pomoca zjawiskelektrycznych. PhD thesis (Uniwersytet Jagiellonski, Krakow).
6. Derbyshire, A.J., Rempel, B., Forbes, A., and Lambert, E.F. (1936). The effects of anesthetics on action potentials in the cerebral cortex of the cat. Am. J. Physiol. Legacy Content *116*, 577–596.
7. Rheinberger, M.B., and Jasper, H.H. (1937). Electrical activity of the cerebral cortex in the unanesthetized cat. Am. J. Physiol. Legacy Content *119*, 186–196.
8. Buzsáki, G., Anastassiou, C.A., and Koch, C. (2012). The origin of extracellular fields and currents—EEG, ECoG, LFP and spikes. Nat. Rev. Neurosci. *13*, 407–420.
9. Noda, H., Manohar, S., and Adey, W.R. (1969). Correlated firing of hippocampal neuron pairs in sleep and wakefulness. Exp. Neurol. *24*, 232–247.
10. Noda, H., and Adey, W.R. (1970). Firing of neuron pairs in cat association cortex during sleep and wakefulness. J. Neurophysiol. *33*, 672–684.
11. Creutzfeldt, O., and Jung, R. (1961). Neuronal discharge in the cat's motor cortex during sleep and arousal. In Ciba Foundation Symposium—The Nature of Sleep Novartis Foundation Symposia, G.E.W. Wolstenholme, and M. O'Connor, eds. (John Wiley & Sons), pp. 131–170.
12. Zagha, E., and McCormick, D.A. (2014). Neural control of brain state. Curr. Opin. Neurobiol. *29*, 178–186.
13. Munk, M.H., Roelfsema, P.R., König, P., Engel, A.K., and Singer, W. (1996). Role of reticular activation in the modulation of intracortical synchronization. Science *272*, 271–274.
14. Cohen, M.R., and Maunsell, J.H.R. (2009). Attention improves performance primarily by reducing interneuronal correlations. Nat. Neurosci. *12*, 1594–1600.
15. Mitchell, J.F., Sundberg, K.A., and Reynolds, J.H. (2009). Spatial attention decorrelates intrinsic activity fluctuations in macaque area V4. Neuron *63*, 879–888.
16. Engel, T.A., Steinmetz, N.A., Gieselmann, M.A., Thiele, A., Moore, T., and Boahen, K. (2016). Selective modulation of cortical state during spatial attention. Science *354*, 1140–1144.
17. Averbeck, B.B., Latham, P.E., and Pouget, A. (2006). Neural correlations, population coding and computation. Nat. Rev. Neurosci. *7*, 358–366.
18. Kanitscheider, I., Coen-Cagli, R., and Pouget, A. (2015). Origin of information-limiting noise correlations. Proc. Natl. Acad. Sci. USA *112*, E6973–E6982.
19. Abbott, L.F., and Dayan, P. (1999). The effect of correlated variability on the accuracy of a population code. Neural Comput. *11*, 91–101.
20. Sederberg, A.J., Pala, A., Zheng, H.J.V., He, B.J., and Stanley, G.B. (2019). State-aware detection of sensory stimuli in the cortex of the awake mouse. PLoS Comput. Biol. *15*, e1006716.
21. Goard, M., and Dan, Y. (2009). Basal forebrain activation enhances cortical coding of natural scenes. Nat. Neurosci. *12*, 1444–1449.
22. Marguet, S.L., and Harris, K.D. (2011). State-dependent representation of amplitude-modulated noise stimuli in rat auditory cortex. J. Neurosci. *31*, 6414–6420.
23. Zagha, E., Casale, A.E., Sachdev, R.N.S., McGinley, M.J., and McCormick, D.A. (2013). Motor cortex feedback influences sensory processing by modulating network state. Neuron *79*, 567–578.
24. Pinto, L., Goard, M.J., Estandian, D., Xu, M., Kwan, A.C., Lee, S.-H., Harrison, T.C., Feng, G., and Dan, Y. (2013). Fast modulation of visual perception by basal forebrain cholinergic neurons. Nat. Neurosci. *16*, 1857–1863.
25. Reimer, J., Froudarakis, E., Cadwell, C.R., Yatsenko, D., Denfield, G.H., and Tolias, A.S. (2014). Pupil fluctuations track fast switching of cortical states during quiet wakefulness. Neuron *84*, 355–362.
26. Schölvinck, M.L., Saleem, A.B., Benucci, A., Harris, K.D., and Carandini, M. (2015). Cortical state determines global variability and correlations in visual cortex. J. Neurosci. *35*, 170–178.
27. McGinley, M.J., David, S.V., and McCormick, D.A. (2015). Cortical membrane potential signature of optimal states for sensory signal detection. Neuron *87*, 179–192.
28. Vinck, M., Batista-Brito, R., Knoblich, U., and Cardin, J.A. (2015). Arousal and locomotion make distinct contributions to cortical activity patterns and visual encoding. Neuron *86*, 740–754.
29. Castro-Alamancos, M.A. (2004). Absence of rapid sensory adaptation in neocortex during information processing states. Neuron *41*, 455–464.
30. Shimaoka, D., Steinmetz, N.A., Harris, K.D., and Carandini, M. (2019). The impact of bilateral ongoing activity on evoked responses in mouse cortex. eLife *8*, e43533.
31. Stringer, C., Pachitariu, M., Steinmetz, N., Reddy, C.B., Carandini, M., and Harris, K.D. (2019). Spontaneous behaviors drive multidimensional, brainwide activity. Science *364*, 255.
32. Burgess, C.P., Lak, A., Steinmetz, N.A., Zatka-Haas, P., Bai Reddy, C., Jacobs, E.A.K., Linden, J.F., Paton, J.J., Ranson, A., Schröder, S., et al. (2017). High-yield methods for accurate two-alternative visual psychophysics in head-fixed mice. Cell Rep. *20*, 2513–2524.
33. Sridharan, D., Steinmetz, N.A., Moore, T., and Knudsen, E.I. (2014). Distinguishing bias from sensitivity effects in multialternative detection tasks. J. Vis. *14*, 16.
34. Jun, J.J., Steinmetz, N.A., Siegle, J.H., Denman, D.J., Bauza, M., Barbarits, B., Lee, A.K., Anastassiou, C.A., Andrei, A., Aydın, Ç., et al. (2017). Fully integrated silicon probes for high-density recording of neural activity. Nature *551*, 232–236.

CellPress
OPEN ACCESS

35. Einstein, M.C., Polack, P.-O., Tran, D.T., and Golshani, P. (2017). Visually evoked 3-5 Hz membrane potential oscillations reduce the responsiveness of visual cortex neurons in awake behaving mice. J. Neurosci. *37*, 5084–5098.

36. Speed, A., Del Rosario, J., Burgess, C.P., and Haider, B. (2019). Cortical state fluctuations across layers of V1 during visual spatial perception. Cell Rep. *26*, 2868–2874.e3.

37. Senzai, Y., Fernandez-Ruiz, A., and Buzsáki, G. (2019). Layer-specific physiological features and interlaminar interactions in the primary visual cortex of the mouse. Neuron *101*, 500–513.e5.

38. Steinmetz, N.A., Zatka-Haas, P., Carandini, M., and Harris, K.D. (2019). Distributed coding of choice, action and engagement across the mouse brain. Nature *576*, 266–273.

39. Steinmetz, N.A., Buetfering, C., Lecoq, J., Lee, C.R., Peters, A.J., Jacobs, E.A.K., Coen, P., Ollerenshaw, D.R., Valley, M.T., de Vries, S.E.J., et al. (2017). Aberrant cortical activity in multiple GCaMP6-expressing transgenic mouse lines. eNeuro *4*, ENEURO.0207-17.2017.

40. de Gee, J.W., Knapen, T., and Donner, T.H. (2014). Decision-related pupil dilation reflects upcoming choice and individual bias. Proc. Natl. Acad. Sci. USA *111*, E618–E625.

41. Kahneman, D., and Beatty, J. (1966). Pupil diameter and load on memory. Science *154*, 1583–1585.

42. Busse, L., Cardin, J.A., Chiappe, M.E., Halassa, M.M., McGinley, M.J., Yamashita, T., and Saleem, A.B. (2017). Sensation during active behaviors. J. Neurosci. *37*, 10826–10834.

43. Fries, P., Roelfsema, P.R., Engel, A.K., König, P., and Singer, W. (1997). Synchronization of oscillatory responses in visual cortex correlates with perception in interocular rivalry. Proc. Natl. Acad. Sci. USA *94*, 12699–12704.

44. Fries, P., Reynolds, J.H., Rorie, A.E., and Desimone, R. (2001). Modulation of oscillatory neuronal synchronization by selective visual attention. Science *291*, 1560–1563.

45. Rohenkohl, G., Bosman, C.A., and Fries, P. (2018). Gamma synchronization between V1 and V4 improves behavioral performance. Neuron *100*, 953–963.e3.

46. Bosman, C.A., Schoffelen, J.-M., Brunet, N., Oostenveld, R., Bastos, A.M., Womelsdorf, T., Rubehn, B., Stieglitz, T., De Weerd, P., and Fries, P. (2012). Attentional stimulus selection through selective synchronization between monkey visual areas. Neuron *75*, 875–888.

47. Engel, A.K., Fries, P., and Singer, W. (2001). Dynamic predictions: oscillations and synchrony in top-down processing. Nat. Rev. Neurosci. *2*, 704–716.

48. Vanhatalo, S., Palva, J.M., Holmes, M.D., Miller, J.W., Voipio, J., and Kaila, K. (2004). Infraslow oscillations modulate excitability and interictal epileptic activity in the human cortex during sleep. Proc. Natl. Acad. Sci. USA *101*, 5053–5057.

49. Mohajerani, M.H., McVea, D.A., Fingas, M., and Murphy, T.H. (2010). Mirrored bilateral slow-wave cortical activity within local circuits revealed by fast bihemispheric voltage-sensitive dye imaging in anesthetized and awake mice. J. Neurosci. *30*, 3745–3751.

50. Mohajerani, M.H., Chan, A.W., Mohsenvand, M., LeDue, J., Liu, R., McVea, D.A., Boyd, J.D., Wang, Y.T., Reimers, M., and Murphy, T.H. (2013). Spontaneous cortical activity alternates between motifs defined by regional axonal projections. Nat. Neurosci. *16*, 1426–1435.

51. Carandini, M., Shimaoka, D., Rossi, L.F., Sato, T.K., Benucci, A., and Knöpfel, T. (2015). Imaging the awake visual cortex with a genetically encoded voltage indicator. J. Neurosci. *35*, 53–63.

52. Shimaoka, D., Harris, K.D., and Carandini, M. (2018). Effects of arousal on mouse sensory cortex depend on modality. Cell Rep. *22*, 3160–3167.

53. Beaman, C.B., Eagleman, S.L., and Dragoi, V. (2017). Sensory coding accuracy and perceptual performance are improved during the desynchronized cortical state. Nat. Commun. *8*, 1308.

54. Harris, K.D., and Thiele, A. (2011). Cortical state and attention. Nat. Rev. Neurosci. *12*, 509–523.

55. Ruff, D.A., and Cohen, M.R. (2019). Simultaneous multi-area recordings suggest that attention improves performance by reshaping stimulus representations. Nat. Neurosci. *22*, 1669–1676.

56. McBride, E.G., Lee, S.J., and Callaway, E.M. (2019). Local and global influences of visual spatial selection and locomotion in mouse primary visual cortex. Curr. Biol. *29*, 1592–1605.e5.

57. Coddington, L.T., and Dudman, J.T. (2019). Learning from action: reconsidering movement signaling in midbrain dopamine neuron activity. Neuron *104*, 63–77.

58. Schultz, W. (2016). Reward functions of the basal ganglia. J. Neural Transm. (Vienna) *123*, 679–693.

59. Ongini, E., and Longo, V.G. (1989). Dopamine receptor subtypes and arousal. Int. Rev. Neurobiol. *31*, 239–255.

60. Vanderwolf, C.H., Gutman, M., and Baker, G.B. (1984). Hypothalamic self-stimulation: the role of dopamine and possible relations to neocortical slow wave activity. Behav. Brain Res. *12*, 9–19.

61. Bao, S., Chan, V.T., and Merzenich, M.M. (2001). Cortical remodelling induced by activity of ventral tegmental dopamine neurons. Nature *412*, 79–83.

62. Monti, J.M., and Jantos, H. (2008). The roles of dopamine and serotonin, and of their receptors, in regulating sleep and waking. Prog. Brain Res. *172*, 625–646.

63. Day, J., and Fibiger, H.C. (1993). Dopaminergic regulation of cortical acetylcholine release: effects of dopamine receptor agonists. Neuroscience *54*, 643–648.

64. Hangya, B., Ranade, S.P., Lorenc, M., and Kepecs, A. (2015). Central cholinergic neurons are rapidly recruited by reinforcement feedback. Cell *162*, 1155–1168.

65. Pachitariu, M., Steinmetz, N., Kadir, S., Carandini, M., and Harris, K.D. (2016). Kilosort: realtime spike-sorting for extracellular electrophysiology with hundreds of channels. bioRxiv. https://doi.org/10.1101/061481.

66. Rossant, C., Kadir, S.N., Goodman, D.F.M., Schulman, J., Hunter, M.L.D., Saleem, A.B., Grosmark, A., Belluscio, M., Denfield, G.H., Ecker, A.S., et al. (2016). Spike sorting for large, dense electrode arrays. Nat. Neurosci. *19*, 634–641.

67. Ma, Y., Shaik, M.A., Kim, S.H., Kozberg, M.G., Thibodeaux, D.N., Zhao, H.T., Yu, H., and Hillman, E.M.C. (2016). Wide-field optical mapping of neural activity and brain haemodynamics: considerations and novel approaches. Philos. Trans. R. Soc. Lond. B Biol. Sci. *371*, 20150360.

68. Zatka-Haas, P., Steinmetz, N.A., Carandini, M., and Harris, K.D. (2018). Distinct contributions of mouse cortical areas to visual discrimination. bioRxiv. https://doi.org/10.1101/501627.

69. Pachitariu, M., Steinmetz, N.A., Kadir, S.N., Carandini, M., and Harris, K.D. (2016). Fast and accurate spike sorting of high-channel count probes with KiloSort. In NIPS Proceedings: Neural Information Processing Systems (NIPS 2016), D.D. Lee, M. Sugiyama, U.V. Luxburg, I. Guyon, and R. Garnett, eds. (Neural Information Systems Foundation), pp. 4455–4463.

70. Reimer, J., McGinley, M.J., Liu, Y., Rodenkirch, C., Wang, Q., McCormick, D.A., and Tolias, A.S. (2016). Pupil fluctuations track rapid changes in adrenergic and cholinergic activity in cortex. Nat. Commun. *7*, 13289.

71. Kalatsky, V.A., and Stryker, M.P. (2003). New paradigm for optical imaging: temporally encoded maps of intrinsic signal. Neuron *38*, 529–545.

72. McCulloch, C.E., and Searle, S.R. (2000). Generalized, Linear, and Mixed Models (John Wiley & Sons).

STAR★METHODS

KEY RESOURCES TABLE

REAGENT or RESOURCE	SOURCE	IDENTIFIER
Experimental Models: Organisms/Strains		
Mouse: Ai93	The Jackson Laboratory	Jax #024103
Mouse: Ai94	The Jackson Laboratory	Jax #024104
Mouse: Ai95	The Jackson Laboratory	Jax #024105
Mouse: EMX1-Cre	The Jackson Laboratory	Jax #005628
Mouse: Rasgrf-Cre	The Jackson Laboratory	Jax #022864
Mouse: Camk2a-tTA	The Jackson Laboratory	Jax #007004
Mouse: VGlut1-Cre	The Jackson Laboratory	Jax#023527
Mouse: tetO-G6s	The Jackson Laboratory	Jax #024742
Mouse: Snap25-G6s	The Jackson Laboratory	Jax #025111
Chemicals, Peptides and Recombinant Proteins		
Muscimol	Sigma-Aldrich	G019
Isoflurane	Merial	N/A
Rimadyl	Pfizer	N/A
Dental Cement	Sun Medical, Moriyama, Shiga Japan	N/A
Cyanoacrylate	World Precision Instruments	VetBond
L-type radiopaque polymer	Sun Medical, Moriyama, Shiga Japan	Super-Bond C&B
Deposited Data		
Preprocessed Widefield Imaging Data	figshare: 10.6084/ m9.figshare.13084805	N/A
Preprocessed Neuropixels Data	This paper [38], figshare	N/A
Preprocessed simultaneous widefield imaging and neuropixels data	figshare: 10.6084/ m9.figshare.13118435	N/A
Software and Algorithms		
MATLAB	MathWorks	N/A
KiloSort	https://github.com/ cortex-lab/ Kilosort [65],	N/A
Phy	https://github.com/ kwikteam/phy [66],	N/A
Glmnet	http://web.stanford.edu/~hastie/ glmnet_MATLAB	N/A
Other		
LCD monitor	Iiyama Adafruit	ProLite E1980 LP097QX1
Fresnel lens	Wuxi Bohai Optics	BHPA220-2-5
Speaker	Tucker-Davies Technologies	MF1-M
CMOS camera (for widefield imaging)	PCO	Edge 5.5
Macroscope	Scimedia	THT-FLSP
1.0x condenser lens	Leica	10450028
0.63x objective lens	Leica	10450027
Dichroic mirrors (for directing excitation light)	Semrock	FF506-Di03,
	Semrock	FF495-Di03-50x70
	Chroma	T425lpxr

(*Continued on next page*)

Continued

REAGENT or RESOURCE	SOURCE	IDENTIFIER
Bandpass filter	Semrock	FF01-482/35-25
470nm LEDs (for GCaMP excitation)	Brain Vision, Cairn OptoLED	LEX2-B P1110/002/000
528nm miniLEDs (for intrinsic imaging)	Thorlabs	LED528EHP
Dichroic mirror (for emitted fluorescence)	Semrock	FF593-Di03
Emission filters	Semrock	FF01-543/50-25
	Edmunds	525/50-55 (86-963)
Excitation filters	Semrock	FF01-466/40-25
	Chroma	ET405-20x
Optical fiber	Cairn	P135/015/003
Neuropixels probes	[34]	Phase 3A Option 3
Micromanipulator	Sensapex Inc.	uMP-4
Infrared LED for pupil tracking	Mightex	SLS-0208A
	Thorlabs	LEDD1B
Camera for pupil tracking	The Imaging Source	DMK 23U618
Camera lens for pupil tracking	Thorlabs	MVL7000
Ophtalmic gel	Alcon	Viscotears Liquid Gel
UV Cement	Norland Optical Adhesives	#81
LED UV Curing System	Thorlabs	CS2010
3D printer	Ultimaker B.V.	Ultimaker 2+,
Steering wheel set-up	[32], UCL CortexLab: [https://ucl.ac.uk/ cortexlab/tools/wheel]	N/A

RESOURCE AVAILABILITY

Lead contact

Further information and requests for resources should be directed to the Lead Contact, Kenneth D. Harris (kenneth.harris@ucl.ac.uk).

Materials availability

This study did not generate new unique reagents.

Data and Code availability

The widefield imaging data generated in this study have been deposited on figshare: [https://doi.org/10.6084/m9.figshare.13084805], in SVD-compressed format (see Method Details). The electrophysiology data from the behavioral experiments is available on figshare (https://figshare.com/articles/steinmetz/9598406). The simultaneous widefield imaging and electrophysiology data have been deposited on figshare: [https://doi.org/10.6084/m9.figshare.13118435].

The code generated for the analysis and visualization of the data is available on GitHub (https://github.com/eakjacobs/Jacobs_et_al_CurrentBiology).

EXPERIMENTAL MODEL AND SUBJECT DETAILS

All experiments were conducted according to the UK Animals Scientific Procedures Act (1986), under personal and project licenses released by the Home Office following appropriate ethics review.

Animals

All animals were on a normal daylight cycle (8am - 8 pm), had access to an exercise wheel in their home cage, and were co-housed whenever possible (2-3 mice per cage; females were always co-housed, males were only co-housed when they were littermates).

The mice came from a variety of genotypes, and in main-text plots (Figures 1, 2, 3, 4, 5, 6, and 7) the mouse line is indicated by symbol color, while glyph shapes represent individual mice. Animals were offspring of double or triple transgenic crosses (males: n = 7, females: n = 9), expressing either GCaMP6f or GCaMP6s in cortical excitatory neurons under the following drivers (color code indicated to the right):

- Ai93; Emx1-Cre; Camk2a-tTa (n = 7, Emerald green)
- Ai94; Emx1-Cre; Camk2a-tTa (n = 1, Cyan)
- Ai94; Rasgrf-Cre; Camk2a-tTa (n = 1, Lavender)
- Ai95; VGlut1-Cre (n = 2, Navy)
- tetO-G6s; Camk2a-tTa (n = 3, Magenta)
- Snap25-G6s (n = 2, Purple)

Our main results held for all genotypes, including lines which can exhibit interictal activity [39].

Surgery

Mice underwent surgery at the age of 8-10 weeks. They were anesthetized with 2% isoflurane in oxygen, body temperature was kept at 37°C, and analgesia was provided by subcutaneous injection of Rimadyl (1ml/0.1kg). The eyes were protected with ophthalmic gel.

In unilaterally imaged animals, the temporalis muscle was detached unilaterally to expose auditory cortex on the left hemisphere. The skull was thinned above visual, auditory and posterior somatosensory cortex using a scalpel until the external table and diploe of the bone were removed. A metal head-plate with a circular opening above posterior cortex was fixed to the cranium with dental cement and a 8mm coverslip was then secured above the thinned skull using UV cement with a LED UV Curing System.

In bilaterally imaged animals, the skull was left intact and a clear skull cap implantation following the method of Steinmetz et al. [39] was used. A light-isolation cone was 3D-printed, implanted surrounding the frontal and parietal bones and attached to the skull with cyanoacrylate. Gaps between the cone and skull were filled with L-type radiopaque polymer. The exposed skull was covered with thin layers of UV cement, and a metal headplate was attached to the skull over the interparietal bone with Super-Bond polymer.

For electrophysiological recordings, craniotomies were performed as described in Steinmetz et al. [38]: mice were anaesthetized with isoflurane while a craniotomy over visual cortex was made with a dental drill or a biopsy punch.

METHOD DETAILS

Behavioral tasks

Mice were trained in one of several variants of a two-alternative choice task [32]. Behavioral training started 1-2 weeks post-surgery, and all animals were handled for habituation prior to head-fixation and training on the tasks. Mice were trained to sit head-fixed in front of an LCD monitor (refresh rate 60Hz), at the bottom of which a MF1 speaker was placed for auditory or auditory distractor experiments. In all but 10 experiments, the monitors were covered with Fresnel lenses to make intensity spatially uniform. The paws of the mice were resting on a steering wheel, which the animals could turn to provide a response in the tasks.

In the basic visual two-alternative forced choice (2AFC) task, a visual Gabor stimulus of varying contrasts appeared randomly in the left or the right visual field. After a brief delay, a go cue indicated the start of the response window. The mouse could move the stimulus on the screen by turning the steering wheel, and was rewarded with water for moving the stimulus to a central location within a response window (1.5-5 s). Incorrect choices (i.e., wheel turns in the wrong direction) or Miss responses (i.e., failure to respond within the allowed time window) resulted in a time-out, which in some mice (10/16) was also signaled with a white noise burst. (The noise burst was dropped in later experiments as it was not necessary for good performance.)

A subset of mice were trained on a visual two-alternative unforced choice (2AUC) version of the task, which contained zero contrast trials for which the animals were required to keep still during the response window in order to receive a reward [32, 33]. In a further subset of these mice, stimuli could be presented on both sides, and the animals were rewarded for moving the higher contrast stimulus to the center, or at random if the contrasts were equal.

In the auditory 2AFC task, low or high frequency tone trains (8 or 15 kHz, respectively) were presented from the speaker directly in front of the mice, and the movement of the wheel was coupled to changes in the tonal frequency of the tone pips. The aim of the task was to bring the tone frequency to the mid-frequency (11 kHz), which was also presented as a go-cue.

The auditory distractor task was identical to the visual 2AFC task, but with irrelevant auditory stimuli presented simultaneously with the visual stimuli. The auditory stimuli consisted of the same auditory tones as in the auditory task, which also changed in frequency as the wheel was turned. However, low and high frequency tones were inconsistently associated with visual stimuli in different sessions, such that in a given session, low was paired with left and high with right, or vice-versa. Even though the auditory stimuli could have provided information about the stimulus within a given session, the animals did not use this to perform the task; when presented with the auditory stimuli alone, they performed at chance level (data not shown). Mice trained on the auditory distractor task had not previously learned the auditory 2AFC task.

To enable analysis of cortical state prior to trial onset, all trials started with a pre-trial baseline of 1-5 s. For the stimulus to appear, the animals had to remain quiescent (keep the wheel still) for 0.5-2 s; early movement lead to a delay in stimulus appearance. Some animals were also trained to keep still for 0.3-0.8 s when the stimulus appeared and wait for a go cue to give their response. In the visual tasks, the go cue consisted of either a tone or a high contrast visual Gabor stimulus at the center of the screen. The modality of the cue did not affect the behavior or the results presented, therefore these tasks were analyzed together. In the auditory and auditory distractor tasks, the go cue consisted of a tone (consisting of the target frequency in the auditory task).

Psychometric curves were generated with the same generalized linear model as in Burgess et al. [32] (see Equations 1–3 in [32]).

Widefield imaging

To correct for hemodynamic artifacts, we used alternate-frame illumination [67]. GCaMP6 fluorescence was excited with a blue LED (470nm), while on alternate frames a green or violet LED was used to measure a calcium-independent hemodynamic signal. Imaging was performed at acquisition rates of 35-50 Hz per color, 10-19ms exposures, with 2x2 or 4x4 binning using a CMOS camera and a macroscope with 1.0x condenser lens and 0.63x objective lens.

Imaging was conducted at two rigs with similar set-ups. At the first set-up, the excitation light was diverted to the brain via a dichroic mirror and passed through a bandpass filter. The green light for capturing the hemodynamic signal was provided by a ring-illuminator containing 5-6 miniLEDs, driven with a LEDD1B driver, that was fixed around the objective. The fluorescence emitted by the brain passed through a dichroic mirror and an emission filter.

At the second set-up (see also [68]), the excitation light passed through an excitation filter, a dichroic (425nm), and 3mm-core optical fiber, then reflected off another dichroic (495nm) to the brain. To capture the hemodynamic signal, the light was passed through a violet excitation filter (405nm) on every other frame. Light from the brain passed through a second dichroic and emission filter to the camera.

Dimensionality reduction

Widefield movie data were compressed and denoised using the singular value decomposition (SVD). All analyses were conducted directly on the SVD-transformed data, allowing much faster execution times that would be required to process the full-pixel movies. Code for such analyses is freely available at https://github.com/cortexlab/widefield.

To compress the data, first, the 3D stack was reshaped into a 2D matrix S of dimensions p x t, where p is the number of pixels and t is the number of time points. Then, we performed SVD of S:

$$S = A\Lambda B^T$$

The physiological spatiotemporal dynamics of the data were fully captured with the top 500 singular values, with lower components encoding only noise. Therefore all the presented analyzes were performed using the top 500 singular values. Each pixel was expressed as a linear combination of the first 500 temporal components of ΛB^T, which we called V; weighted by the corresponding spatial matrix U, consisting of the first 500 spatial components of A.

Hemodynamic correction

Hemodynamic correction was performed by subtracting a multiple of the calcium-independent signal from the GCaMP signal. The multiple used was allowed to vary between pixels; to estimate this multiple, both GCaMP and hemodynamic signals were first linearly de-trended and high-pass filtered above 0.01Hz, and then bandpass filtered in the frequency range corresponding to the heart beat (9-13Hz), where hemodynamic artifacts are strongest. The optimal multiple was estimated by linear regression; pixel-wise multiplication and subtraction was performed in the SVD domain to allow faster analysis. The code for this method can be found at https://github.com/cortexlab/widefield/blob/master/core/HemoCorrectLocal.m.

Hemodynamic correction was performed on all data except for data from 3 early animals that were imaged using blue illumination only. However, because later analyses indicated that spectral analysis results were not affected by hemodynamic artifacts, the data from these 3 animals was also included in the paper. For the baseline fluorescence analysis however (Figure S4), these animals were excluded.

Electrophysiological recordings

Electrophysiological recordings were performed as in Steinmetz et al. [38]. Briefly, recordings were made with Neuropixels Phase 3A probes [34] affixed to metal rods and moved with micromanipulators. Probes were advanced through saline based agar that covered the craniotomies and through the dura, then lowered to their final position at ~10 μm/sec. Electrodes were allowed to settle for ~15 min before starting recording. Spikes were isolated from the action potential band (high-pass filtered over 300Hz) by subtracting the median across all channels to de-noise and spike-sorting using KiloSort [69]. Units were manually inspected and any units representing noise were removed. Units within the cortex were identified by depth and by inspection of physiological signatures. All spikes within the cortex were then combined to yield one cortical multiunit signal.

In experiments with simultaneous electrophysiology and widefield imaging, light-induced artifacts were reduced by ramping the widefield illumination for 1ms on the light onset and offset. For the local field potential (LFP) analysis, light-induced artifacts were removed from the LFP signal by aligning the LFP trace to all light onsets of a single color, subtracting the baseline (the sample before each light onset), creating a running median of 500 light pulses, and subtracting the resulting trace from the raw data.

Coherence and spectral correlation was then calculated between local field potential or multiunit activity (MUA) and cortical fluorescence within the craniotomy.

For inactivation experiments, muscimol (5 mM in ACSF) was applied topically by placing muscimol-soaked gelfoam in a craniotomy for 40 minutes, with additional ACSF applied at 20 minutes to prevent drying.

In the behavioral experiments, the visual task was the same as during the widefield imaging, and 4 of the 5 mice had previously performed the same task during widefield imaging.

Eye tracking

Neural recordings were paired with eye tracking recordings in all but 10 datasets. One of the eyes (usually the eye contralateral to the imaged hemisphere in unilaterally imaged recordings) was illuminated with an infrared LED and recorded using a CCD camera with an infrared filter and a zoom lens. The videos were recorded with MATLAB's Image Acquisition Toolbox. Pupil size and position were computed following the methods from Burgess et al. [32]. All obtained pupil traces were further processed following the methods of Reimer et al. [70].

Behavioral measurements

Trials were divided into four classes. They were classified as "Choice" trials if the animal provided a choice ("Correct" or "Incorrect") within the response window. They were classified as "Miss" trials if the animal failed to provide a response within the response window, when a response was required to obtain a reward. They were considered "Correct Reject" trials when the animal correctly provided a "no-go" response by withholding a response throughout the response window. They were considered "False Alarm" trials when the animal incorrectly moved the wheel during trials that required a "no-go" response. The % Correct, Incorrect and Miss in Figure 1 was computed using a sliding window over 10 trials. Reaction times in Figure S5 were defined as the interval of time between go-cue onset and response time. Since reaction time varied between stimuli, we computed the average reaction time per stimulus, subtracted this average per trial per contrast, and used the obtained residuals for computing the Pearson correlation with power.

The baseline period was defined as the inter-trial interval (ITI) preceding the stimulus onset at each trial. Quiescent periods were defined as the end of the ITI during which no movement of the wheel was detected. The body of the mice was restrained by the behavioral tube they were sitting in, making large overt movements other than steering the wheel impossible. Trials with quiescent periods of less than 0.7 s were excluded from analysis.

QUANTIFICATION AND STATISTICAL ANALYSIS

Stimulus-triggered responses

Trials containing 50% or higher contrast on the right visual field were averaged and baseline subtracted at time 0 of stimulus onset. The map in Figure 2H consists of the frame at t = 70-80ms post stimulus onset subtracted by the previous frame. This method was chosen as it revealed the cleanest stimulus response due to the slow dynamics of GCaMP6s.

Power maps

As explained under 'Dimensionality reduction', for a given pixel n, the fluorescence over time was represented by $f_n(t) = U_n \cdot V = \sum_{i=1}^{500} U_{ni} V_{it}$, where U_n is the row within U corresponding to pixel n. Therefore, the Fourier transform of $f_n(t)$ was calculated as

$$\widehat{f_n}(\omega) = \sum_{i=1}^{500} U_{ni} \widehat{V}(\omega),$$

where ^ denotes the Fourier transform. To compute the power in the frequency band of interest 3-6Hz, we calculated $\sum_{\omega=3Hz}^{6Hz} \widehat{f_n}(\omega) \widehat{f_n}^*(\omega)$. The power spectrum for all pixels could thus be efficiently computed using matrix multiplication, at least an order of magnitude faster than without the SVD compression. Finally, this was reshaped into a 2-dimensional 'Power map', $P(x,y)$, where x and y are spatial dimensions.

We computed these power maps during the quiescent period for each trial separately, then computed the average power for Choice and Miss conditions:

$$P_{choice}(x,y) = \frac{1}{Zchoice} \sum_{i=1}^{Zchoice} P(x,y)$$

$$P_{Miss}(x,y) = \frac{1}{Zneglect} \sum_{i=1}^{Zneglect} P(x,y)$$

The power difference maps were then computed as follows:

$$P_{Diff}(x,y) = 10 \cdot \log_{10} \left(\frac{Pchoice}{Pneglect} \right)$$

The multiplication by 10 is applied to turn the power ratios into units in decibels (dB).

The same principle applied for computing power difference maps between Correct and Incorrect Choice trials, and so on.

In the power difference maps shown in the figures, pixels with average power (over time) below the 20th percentile were set to black. This procedure effectively masks pixels outside the brain.

ROI selection

Outlines of visual and auditory cortices were identified in each mouse by sensory stimulation using sweeping bars [71] for visual cortex, and repeated pure tone pips of different frequencies for auditory cortex [52].

The region of interest (ROI) in visual cortex (VIS) was chosen as the center of the average stimulus response to contralateral stimuli within the visual task. The ROI in auditory cortex (AUD) was based on the auditory cortex maps obtained by passive stimulation. The responses to different frequencies were averaged and the ROI was selected from the area with the highest mean response and which was responsive to the frequencies used within the auditory task.

The position of somatosensory cortex was estimated stereotaxically, and confirmed functionally by manually inspecting imaging activity during whisking and movement. The ROI in somatosensory cortex (SS) was chosen from within the area that was estimated to be the barrel cortex.

The ROI in retrosplenial cortex (RSP) was estimated stereotaxically and always chosen from posterior RSP as this was the visible part of RSP in the unilateral imaging experiments. The secondary motor cortex ROI (MO) was estimated stereotaxically.

The individual patterns of vasculature in each mouse were used as additional guidance to place the ROIs as consistently as possible across experiments from the same individuals. ROIs in the bilateral imaging experiments were chosen from the left hemisphere to be consistent with the unilateral imaging experiments.

The power for each ROI was estimated as the 'power map' value at a single pixel in the ROI center; in practice however, this averages a signal from a slightly larger region due to the spatial smoothing resulting from the SVD representation.

MUA and LFP power

To directly measure the amplitude of slow fluctuations in population activity, the recorded MUA firing rate was down-sampled to the same sampling rate as the imaging experiments (35Hz) and then further smoothed with a Gaussian window. The LFP and MUA 3-6Hz band power was computed during the quiescent period, same as in the imaging experiments, and the results of this were analyzed in an identical manner to the imaging results.

Fano factor

The fano factor was computed for each trial and we used the following standard formula:

$F = var(X)/mean(X)$, where X refers to the spike count during the same time window as was used for computing pre-stimulus quiescent period power.

Decoder analysis

To decode the contralateral stimulus from V1 population activity, we trained a decoder using the cvglmnet MATLAB package (http://www.stanford.edu/~hastie/glmnet_matlab/), to perform logistic regression using L2 normalization and 5-fold cross validation. Only recordings for which the visual receptive fields of the recorded neurons were topographically aligned with the contralateral stimulus were used for these analyses. For each trial, a population activity vector was computed from the spike counts of all cells in a time bin of duration 100 ms; the optimal window was found by search (100 ms resolution) to be 50-150 ms after stimulus onset. To ensure equal training data for each two conditions being compared (Choice versus Miss, Low versus High Power, or Low versus High Fano factor), the data was subsampled such that there were equal trial numbers for each contrast (25%, 50% and 100%) per decoder condition. For example, in case there were 26 50% contralateral contrast Choice trials and 18 50% contrast Miss trials, we randomly selected 18 of the 26 50% contralateral contrast Choice trials. To define trials as Choice and Miss, the same criteria were used as in all other analysis. To divide trials into groups of low or high 3-6Hz Power or Fano factor, we used trials below and above the median, respectively. The numbers of trials in each contrast condition were equalized in the same way for these comparisons as for the Choice versus Miss comparison.

Power percentiles

In the analysis of behavior (% Miss, Incorrect or False Alarm; Figures 2, 3, 4, S4, and S6) or Fano factor (Figure 2) as a function of power, the power values corresponding to 20%, 40%, 60% and 80% of maximum power for each ROI in a given session were calculated using MATLAB's prctile function. This provided 5 power percent values, and each trial was classified as belonging to the first (below 20%), second (between 20% and 40%), third (between 40%–60%), fourth (between 60%–80%) and fifth (above 80%) percentile. All trials belonging to a given percentile were further subdivided into Choice versus Miss and Correct versus Incorrect for trials containing contrasts, and Correct Reject versus False Alarm for trials containing no contrasts. Equal contrast trials were excluded in the Correct versus Incorrect comparison, as these trials were rewarded randomly and thus were not informative in terms of the effect of power on performance. For example, if $N_{trials} = 50$ in the first percentile, out of which $N_{contrast\,=}$ 40 trials with contrasts and $N_{zero} = 10$ trials with zero contrast; within $N_{contrast}$ there were $N_{choice} = 36$ Choice trials and $N_{miss} = 4$ Miss trials, within N_{choice} there were $N_{nonequalContrast} = 35$ non-equal contrast trials, of which $N_{correct} = 28$ Correct trials and $N_{incorrect} = 7$ Incorrect trials; and within N_{zero} there were $N_{reject} = 6$ Correct Reject trials and $N_{falsealarm} = 4$ False Alarm trials, then this would have given: $N_{choice}/ N_{contrast}*100 = 90\%$ Choice and $N_{miss}/ N_{contrast}*100 = 10\%$ Miss; $N_{correct}/ N_{nonequalContrast}*100 = 80\%$ Correct and $Nin_{correct}/ N_{nonequalContrast}*100 = 20\%$ Incorrect; and $N_{reject}/ N_{zero}*100 = 60\%$ Correct Reject and $N_{falsealarm}/ N_{zero}*100 = 40\%$ False Alarm rates for the first percentile. This computation was repeated for all percentiles; for all ROIs of interest and for all datasets. The overall % Miss, % Incorrect and % False Alarm probabilities were then calculated as the average across all datasets for each percentile. Statistical significance was assessed as described in Statistics.

Statistics

To allow for differences between mice or between recording sessions for a single mouse, which would violate the assumption of independent variates, all statistical tests were conducted using nested mixed effects models [72] as this enables testing for significance of one factor while allowing random variability in others. Trial type (Choice versus Miss, Correct Choice versus Incorrect Choice, etc) and cortical area (VIS, SS, MO, RSP and AUD) were modeled as fixed effects, and we tested whether these variables had an effect on low-frequency power. Session, mouse, and genotype were treated as nested random effects that interacted with area, to allow for potential area-dependent differences in power between genotypes; potential area-dependent differences in power between individual mice of a single genotype; and potential area-dependent variability in power between sessions of a single mouse. We first tested for a main effect of trial type and brain area, and if it was present we then tested for interactions. Models were fit using maximum likelihood fitting with unconstrained covariance matrices using MATLAB's fitlme function with default parameters; the models used in this command were "power ~ cond + area + (area|genotype) + (area|subject:genotype) + (area|session:subject:genotype)" without interaction, and "power ~ cond * area + (area|genotype) + (area|subject:genotype) + (area|session:subject:genotype)" with interaction. We validated this statistical approach by simulating data with random differences between mice, sessions and genotypes, but no effect of trial type, and verifying that the test did not report a significant effect. Datasets that had fewer than 10 trials in a behavioral condition that was being compared to another (Choice versus Miss, Correct versus Incorrect) were excluded from analysis.

To assess whether power percentile had a significant effect on behavior, we fit a generalized linear mixed model separately for each ROI with a random effect of experimental session and a fixed effect of power percentile, and a logistic link function. For this, we used MATLAB's fitglme function with model "choice ~ 1 + powerPercentile + (1 | Session)." A significantly different effect between ROIs was considered when the 95% confidence intervals did not overlap. To assess whether there was a significant difference in decoder performance between conditions, we used the same approach: MATLAB's fitlme function with model "decoding ~ cond + (1|subject) + (1|session:subject)," where "decoding" consists of the decoder performance, and "cond" was either Choice-Miss, or Desynchronized-Synchronized (following the definitions given under Decoder Analysis).

In the pupil analysis, data were analyzed with one-way analysis of covariance models (ANOCOVA) fitting separate but parallel lines to the data per behavioral condition. Multiple comparisons were adjusted using Tukey's honest significant difference criterion.

Current Biology, Volume *30*

Supplemental Information

Cortical State Fluctuations

during Sensory Decision Making

Elina A.K. Jacobs, Nicholas A. Steinmetz, Andrew J. Peters, Matteo Carandini, and Kenneth D. Harris

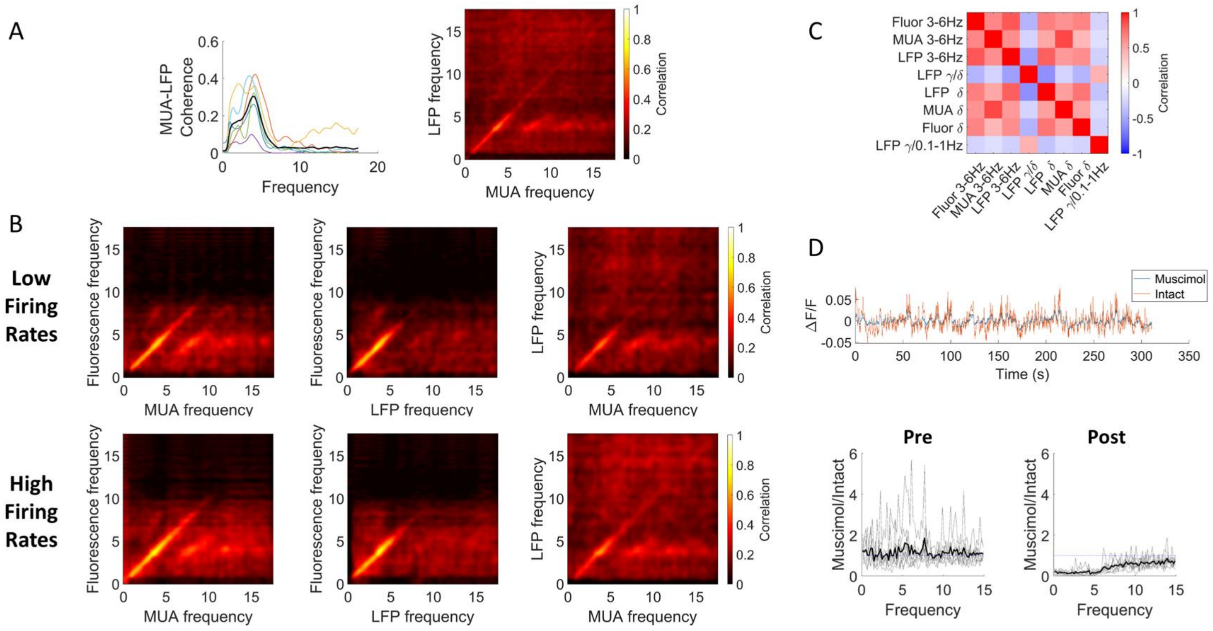

Figure S1. Low-frequency widefield power correlations with electrophysiological measures of power. Related to Figure 2. A. Left: coherence correlation between MUA and LFP. Black trace = average, colored traces = individual experiments (n = 6 experiments from 6 animals). Coherence of LFP with MUA is smaller than coherence of widefield fluorescence with MUA (peak of 0.31 vs. 0.56; $p<0.05$, t-test; cf. Figure 2C), likely reflecting non-local contributions to the LFP signal. Right: cross-frequency power correlation between MUA and LFP. **B.** Power measured by widefield imaging is strongly correlated with power measured by electrophysiology, and does not reflect changes in firing rate. We divided each dataset into low and high firing rate periods (epochs of 2 s or longer consistently above or below the median MUA firing rate). The correlations between widefield and MUA power, widefield and LFP power, and MUA and LFP power all remain comparable when restricted to periods of low (top row) and high (bottom row) firing rates. **C.** Correlations between widefield fluorescence, MUA, and LFP signals across multiple frequency bands, together with LFP power ratios (gamma to delta; gamma to ultraslow). Each square represents the average correlation across six experiments. Note the positive correlation of power in all low-frequency bands and modalities, and its inverse correlation to the LFP γ/δ and γ/infraslow ratios (sometimes used as measure of desynchronization). δ = 1-4Hz, γ = 20-80Hz. **D.** Local application of muscimol ablates low frequency power in the widefield signal. Top: Example widefield trace of cortical activity with and without muscimol application. Blue trace indicates the signal from the muscimol-treated cortical region, brown trace indicates the signal from the corresponding contralateral untreated cortical region. Bottom: ratio of muscimol-treated to control (intact) power spectra. Left: Before muscimol application, the ratio hovers around 1, indicating that the power spectra from the two cortical regions are comparable across frequencies. Right: After muscimol treatment, the ratio drops to close to zero at frequencies below ~8Hz, indicating a large drop in widefield power after local cortical activity has been inhibited by muscimol, indicating that the majority of the widefield signal reflects local spiking activity.

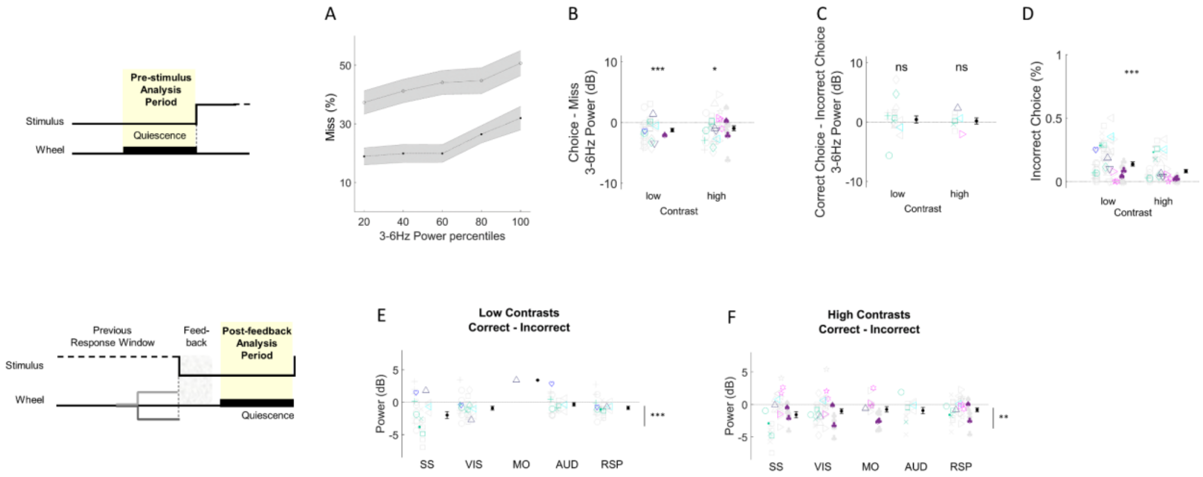

Figure S2. Power differences between trial types do not depend on contrast. Related to Figures 2 and 6. **A-D:** Behavior and power differences per contrast condition. **A.** Average percent Miss trials in different 3-6Hz power percentiles for high (filled circles) and low (open circles) contrasts. Lower contrasts are generally more likely to be 'Missed', but high contrasts also get Missed, and increasing 3-6Hz power equally increases the Miss probability for both low and high contrasts (p<0.001 for both high and low contrasts, generalized linear mixed-effects model). **B**. Summary of 3-6Hz power difference in visual cortex between choice and Miss trials for low and high contrasts. Choice trials are significantly more desynchronized for both contrast conditions (condition main effect p<0.001; contrast main effect p>0.05, nested mixed effects ANOVA). **C**. Same as B but comparing Correct and Incorrect Choice trials (condition main effect p>0.05; contrast main effect p>0.05, nested mixed effects ANOVA). **D**. Summary of percent Incorrect Choice for low and high contrasts across experiments. Animals are significantly more likely to provide an Incorrect Choice for low contrasts (p<0.001, Wilcoxon signed rank test). High contrasts consist of trials including and above 50% contrast, low contrast trials consist of trials below 50% contrast. Contrast comparison trials were included in the high contrast trials as excluding them did not affect the results (data not shown). **E-F:** The effect of reward does not depend on contrast. **E.** Summary difference in 3-6Hz power after Correct and Incorrect low contrast trials. **F.** As E but for high contrast trials. After both low and high contrasts, Correct i.e. rewarded trials show prolonged desynchronization lasting into the quiescent period of the following trial. There was no effect of contrast on the power difference between rewarded (Correct) and non-rewarded (Incorrect) trials (p<0.05, nested mixed ANOVA where session was nested into mouse, session was set a random effect, and the main effects and interactions of session, ROI, response (Correct or Incorrect) and subject were included in the model). ***, p < 0.001; ns, p≥0.05

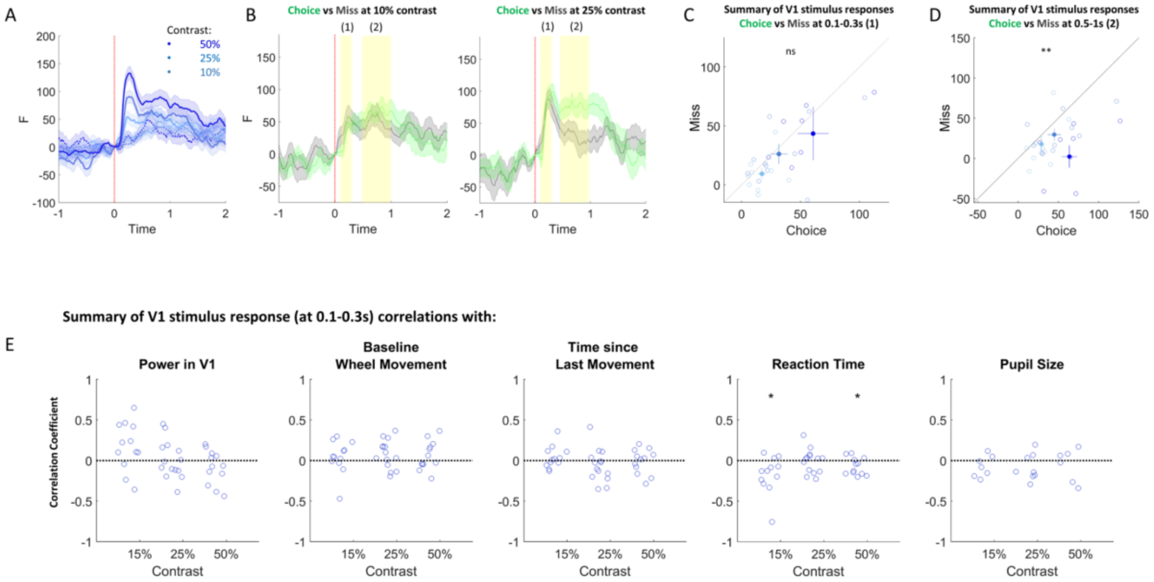

Figure S3. V1 stimulus responses measured by widefield imaging. Related to Figure 2. A. Time course of V1 responses to visual stimuli of different contrasts from an example mouse. **B**. Example time courses for 10% and 25% contrast, split by behavioral condition (Choice and Miss). **C**. Population summary of average amplitudes of V1 responses at 0.1 to 0.3 seconds (highlighted in B as (1)) post stimulus onset. The amplitude was computed as the average fluorescence signal obtained from the time period in (1). Open circles indicate individual datasets, filled circles indicate average per contrast. Colors are the same as indicated in the legend in A. There was no significant difference between Choice and Miss responses (p>0.05, 2-way ANOVA). **D**. Same as C but for a later timepoint (0.5 to 1 seconds post stimulus onset) that overlaps with reaction time onset. **E**. Summary of correlations between V1 amplitudes at the first timepoint (1) and relevant physiological and behavioral factors. Shaded areas in A and B indicated standard error of the mean (SEM); bars in C and D indicate SEM. n = 14 experiments from 4 mice. Only datasets in which no movement was detected during the first analysis window were included, and only contrast conditions with at least 5 trials per behavioral condition were included for each dataset. *, p < 0.05; **, p<0.01; ns, p>0.05

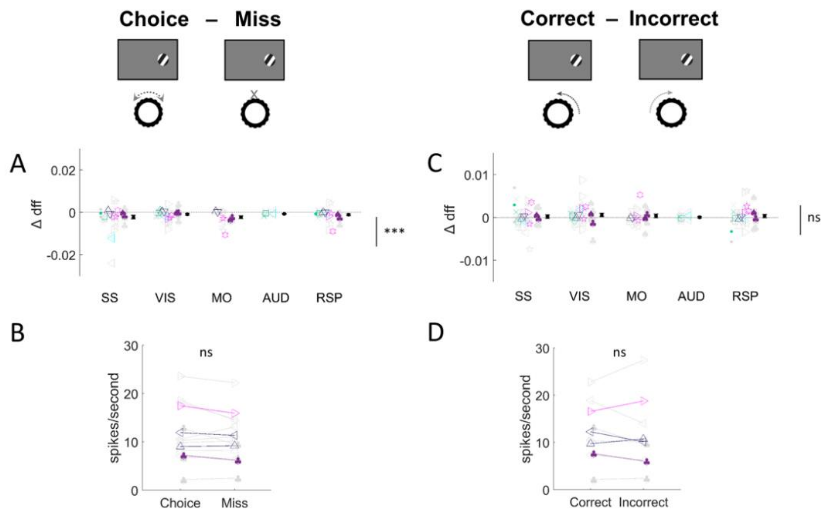

Figure S4. Differences in baseline activity do not explain the effects on 3-6Hz power. Related to Figure 3. A. Choice – Miss difference in dff fluorescence. There is a small but significant difference, with Miss baseline activity increased compared to Choice activity. The decrease in power can therefore not be driven by an increase in activity levels. **B.** Comparison of baseline firing rates from Neuropixels recordings. **C-D.** Similar analysis but comparing Correct and Incorrect trials.

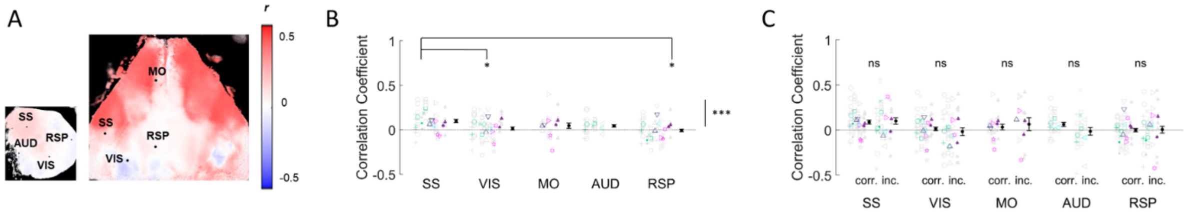

Figure S5. 3-6Hz power correlates with subsequent reaction time. Related to Figure 3. A. Pseudocolor maps showing correlation of reaction time with 3-6Hz power in each pixel. Red indicates a positive correlation: the lower the power, in other words the more desynchronized the cortical state, the faster the reaction time. **B.** Summary of power-reaction time correlations for all experiments. The correlation in somatosensory cortex was significantly stronger than in visual and retrosplenial cortex ($p<0.05$, one-way ANOVA. The significant overall effect was computed using one sample t-test for all correlations). **C.** Same as B but split into Correct (corr.) and Incorrect (inc.) Choice trials. There was no difference in correlation between 3-6Hz power and reaction time between Correct and Incorrect Choice trials in all ROIs, suggesting the correlation reflected a general readiness to respond and no selective effects depending on performance accuracy. *, $p < 0.05$; ***, $p < 0.001$; ns, $p \geq 0.05$

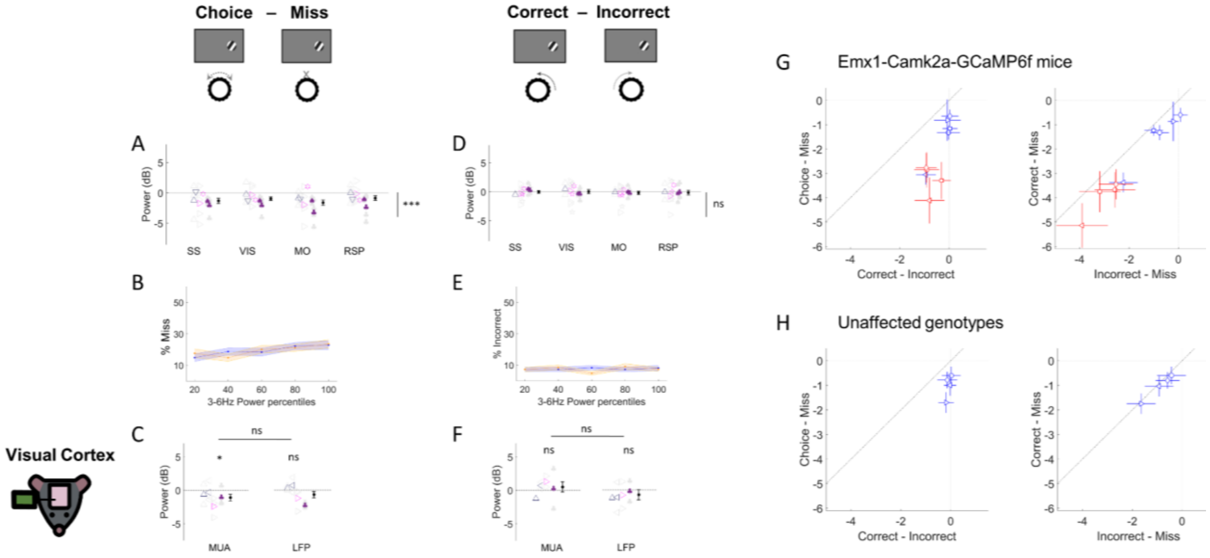

Figure S6. Excluding animals from genotypes that are prone to interictal activity did not change the results, and electrophysiological recordings in visual cortex replicated the same results as obtained by imaging. Related to Figures 3 and 7. A. Summary Choice-Miss 3-6Hz power differences ($p<0.001$, nested mixed ANOVA, n = 21 experiments from 6 animals excluding interictal-prone lines). Similar results were obtained when considering GCaMP6f animals alone (n = 38 experiments from 8 animals, $p<0.001$ nested mixed ANOVA) and GCaMP6s animals alone (n = 20 experiments from 7 animals, $p<0.001$ nested mixed ANOVA) separately. **B.** Percent Miss increases equally with increasing 3-6Hz power in visual (blue, $p<0.01$) and somatosensory (orange, $p<0.05$) cortex. **C.** 3-6Hz power differences in visual cortex as measured from multi-unit activity (MUA) and local field potentials (LFP) from Neuropixels recordings in some of the same animals that had previously been imaged and are shown in A. n = 10 experiments from 4 animals. **D-F.** Similar analysis as A-C but comparing Correct and Incorrect Choices. **E.** Visual cortex (blue): p=0.7, Somatosensory cortex (orange): p =0.9. **G-H.** Task related cortical state differences do not depend on genotype. **G.** Each point shows the power difference (dB) for Choice vs. Miss trials and Correct vs. Incorrect trials, averaged over all subjects, for a specific cortical area in in EMX1-Camk2a-GCaMP6f mice. Blue symbols are for visual task, red for auditory task. Error bars represent standard error of the mean over subjects. **H.** Results from mice of genotypes for which interictal activity has not been reported, in the visual task. *, $p<0.05$; ***, $p < 0.001$; ns, $p\geq0.05$

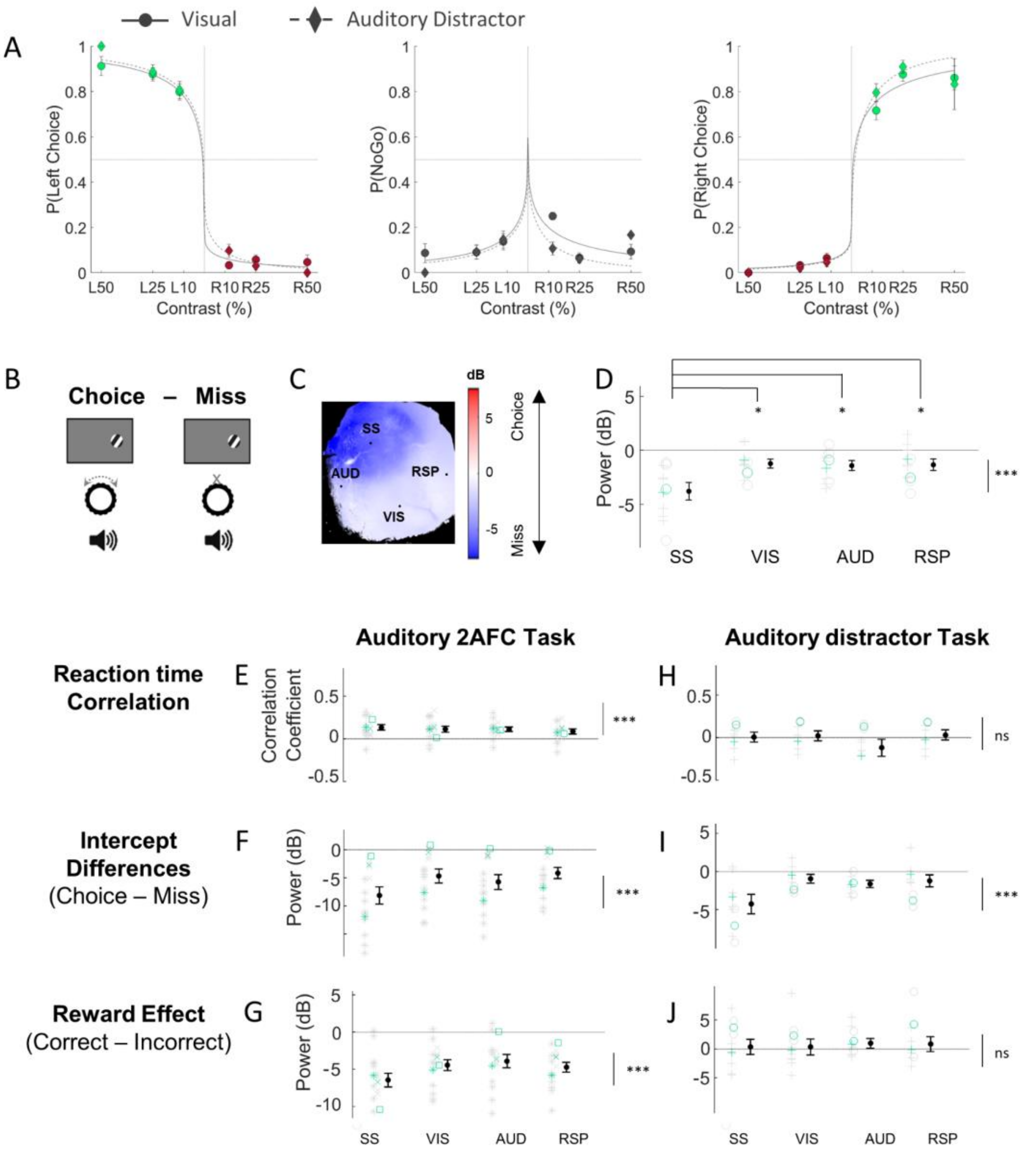

Figure S7. Results from the auditory 2AFC and auditory distractor tasks. Related to Figures 5, 6 and 7. A-D: The auditory distractor task. **A.** Psychometric curves comparing the performance during the normal visual and the auditory distractor task. The auditory distractor task consisted of the visual 2AFC task onto which irrelevant auditory tones were added (the same ones as in the auditory task, but they were inconsistently paired with the visual stimuli so as not to provide any extra information about the stimulus). The mice successfully disregarded the auditory stimuli and performed equally as well in the auditory distractor task as in the normal visual task. **B.** Cartoon illustrating the comparison of Choice and Miss trials in the auditory distractor task. **C.** Pseudocolor map showing the 3-6Hz power difference for each pixel; blue indicates higher power on Miss trials. **D.** Summary of 3-6Hz power differences between Choice and Miss trials for selected ROIs across all experiments in the auditory distractor task (n = 10 experiments from 2 animals). **E-J:** The results regarding reaction time correlation, relationship with pupil, and effect after reward are replicated in the auditory and auditory distractor tasks. *, $p < 0.05$; ***, $p < 0.001$; ns, $p \geq 0.05$
